# Supplementary material for: Doping-induced structural phase transition in cobalt diselenide enables enhanced hydrogen evolution catalysis
Source: Nat Commun. 2018 Jun 28;9:2533. doi: 10.1038/s41467-018-04954-7 (PMC6023930; doi:10.1038/s41467-018-04954-7)
Supplement: Supplementary file 1 — Supplementary Information [file 41467_2018_4954_MOESM1_ESM.pdf]

Supplementary Information

Doping-induced structural phase transition in cobalt diselenide enables enhanced hydrogen evolution catalysis

Zheng et al.

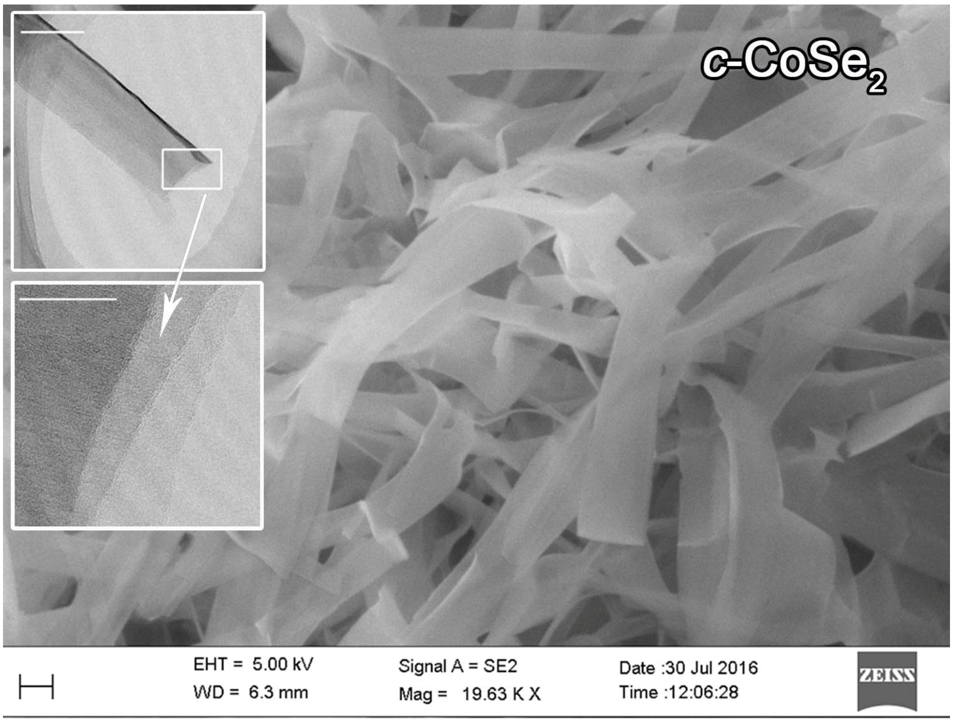

Supplementary Figure 1. Layered *c*-$CoSe_2$ nanobelts. SEM image shows the morphology of *c*-$CoSe_2$ nanobelts with widths of 200-800 nm and lengths up to several micrometers. Scale bar: 200 nm. Insets are TEM and HRTEM images that show well-defined multilayered nanostructure along the thickness direction. Scale bars: 200 nm (up) and 20 nm (down).

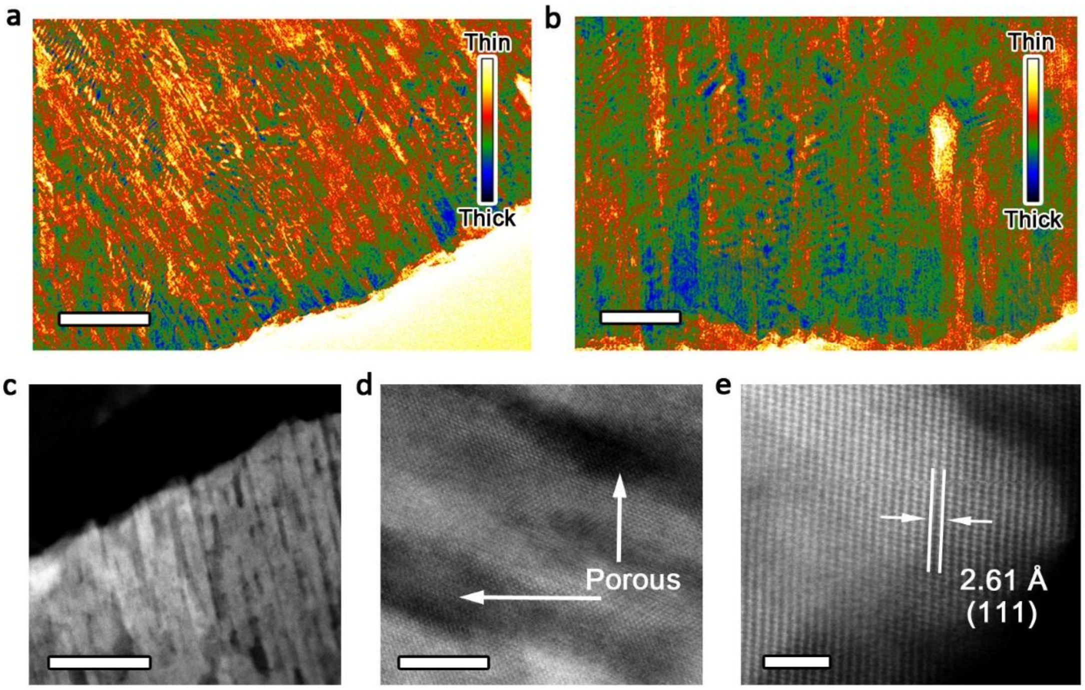

Supplementary Figure 2. HAADF images of *o*-$CoSe_2$|P. a, **b,** False-color HAADF images show the relative thickness of as-prepared *o*-$CoSe_2$|P. Scale bar, 50 nm. **c-e,** HAADF-STEM images of the *o*-$CoSe_2$|P catalyst with different magnifications, which show its porous structure and high-crystalline feature. Scale bars: 50, 10, and 2 nm, respectively.

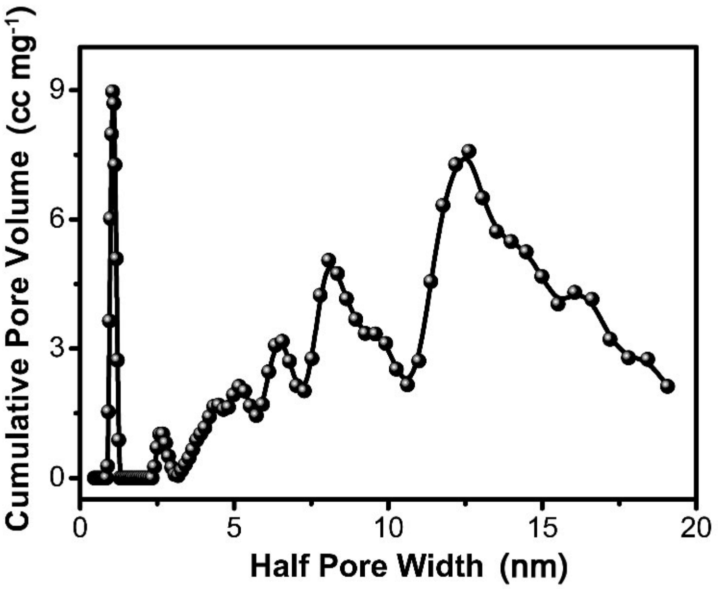

Supplementary Figure 3. The pore size distribution curves of *o*-$CoSe_2$|P. It shows that the *o*-$CoSe_2$|P catalyst possesses a hierarchically porous structure containing multi micropores centered at 1.0 nm and mesopores centered between 6.5 and 15 nm. Such mutiporous structure gives a high surface area, thus rich catalytic active sites for HER catalysis.

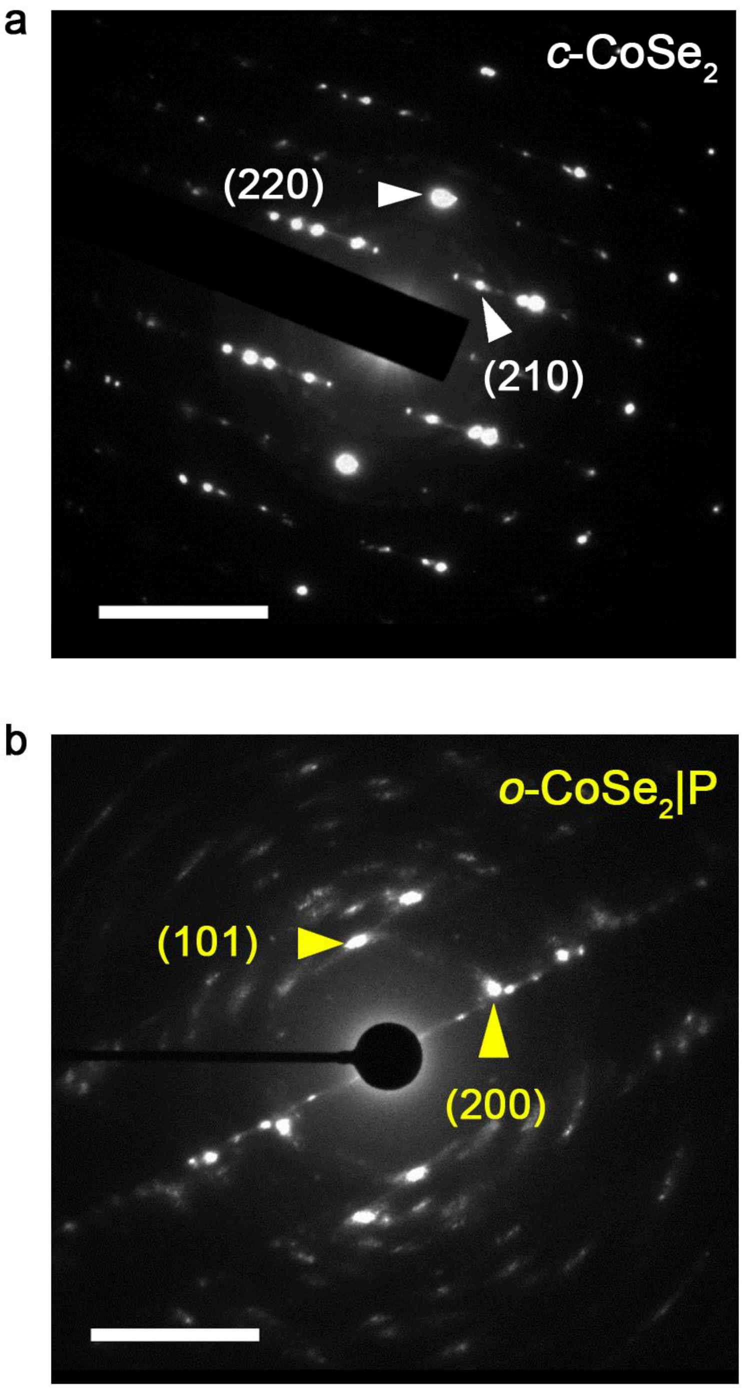

Supplementary Figure 4. Comparison of SAED patterns between *c*-$CoSe_2$ and *o*-$CoSe_2$|P. a, The SAED pattern taken on a typical *c*-$CoSe_2$ nanobelt, which exhibits a cubic structure with single-crystalline character. **b,** The SAED pattern of *o*-$CoSe_2$|P. Owing to the high porosity of *o*-$CoSe_2$|P nanobelt and the lattice mismatch from the participation of P, the SAED pattern of *o*-$CoSe_2$|P displays a quasi-single-crystal feature with slightly stretched diffraction spots. Scale bars: 5 1/nm.

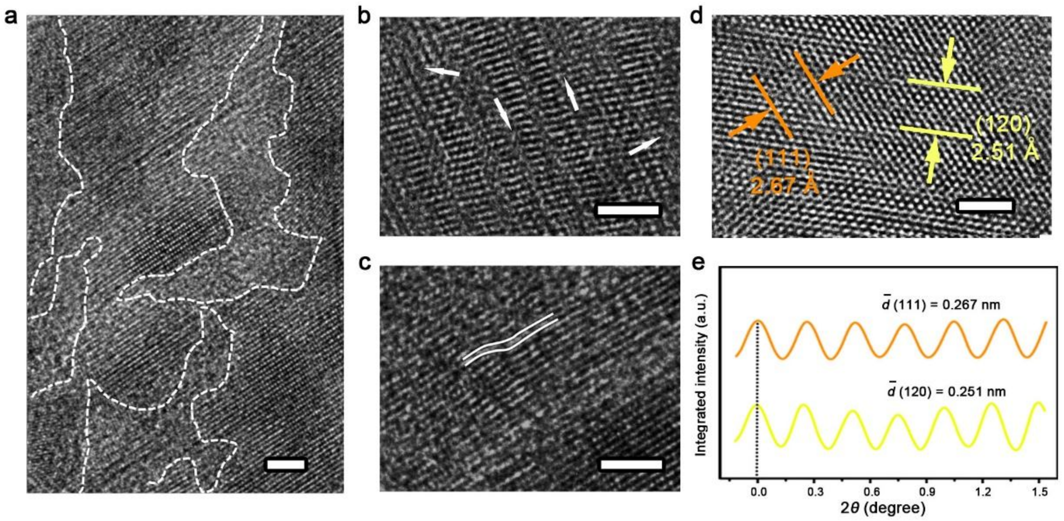

Supplementary Figure 5. High-resolution TEM (HRTEM) images of *o*-CoSe$_2$|P. a-c, HRTEM images of *o*-CoSe$_2$|P shows defects around the pore edges. Scale bars, 2 nm. These defects were caused because of the thermal treatment and bonds rotation during phase transition, which creates more active sites and benefits the HER catalysis. **d, e,** HRTEM image of *o*-CoSe$_2$|P shows a high-crystalline orthorhombic structure. Scale bar, 2 nm. The orange and yellow curves denote the integrated pixel intensities of spacings along (111) and (120) facets, respectively.

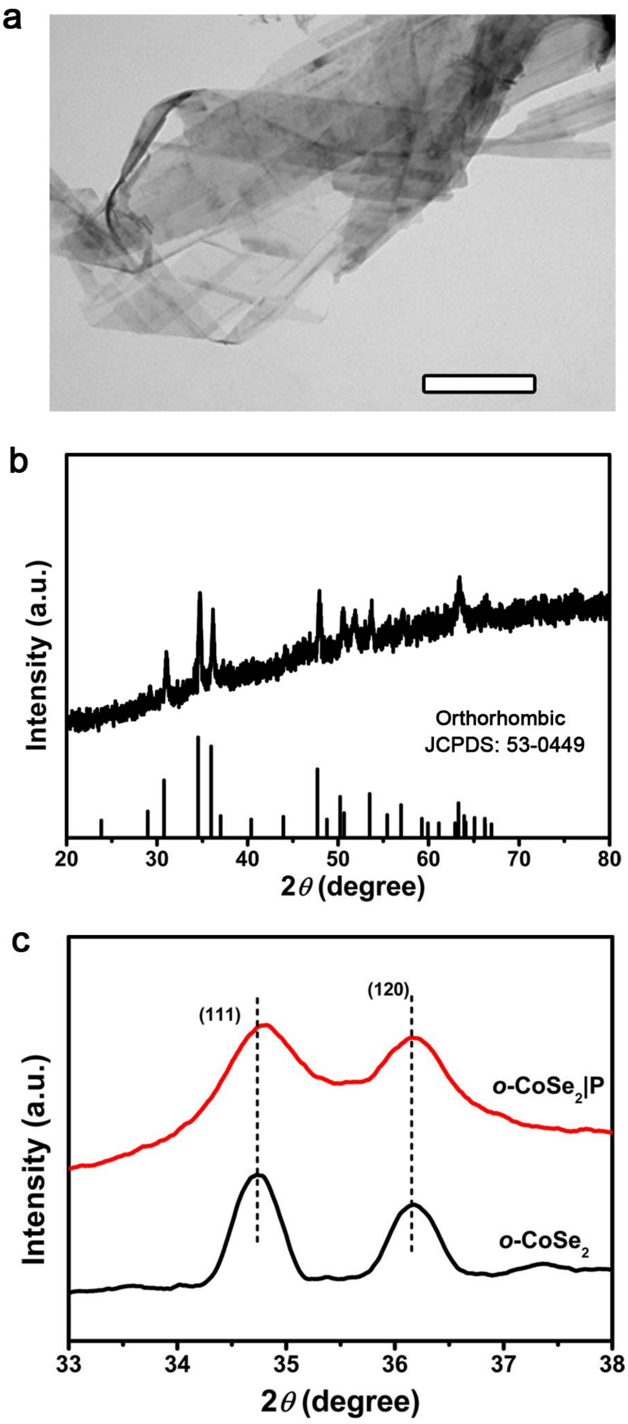

Supplementary Figure 6. XRD patterns of *o*-$CoSe_2$|P and *o*-$CoSe_2$. a, TEM image of the prepared *o*-$CoSe_2$ refer to the previously reported literature[1]. Scale bar, 200 nm; **b,** XRD pattern of *o*-$CoSe_2$ with the standard pattern of the orthorhombic $CoSe_2$ (JCPDS: 53-0449) as reference. **c,** Selected XRD pattern insets of the (111) and (120) diffraction peaks that compare *o*-$CoSe_2$|P (red) and *o*-$CoSe_2$ (black). The peak position of *o*-$CoSe_2$|P shifts to a higher angle indicating smaller P atoms that replace partial bigger Se atoms.

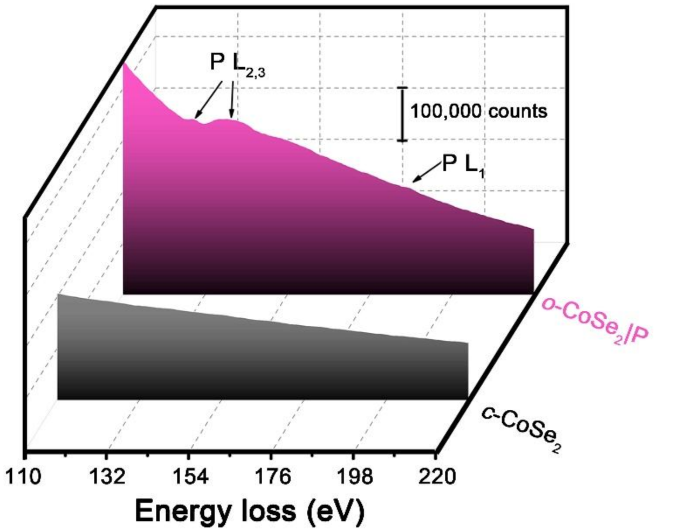

Supplementary Figure 7. EELS spectra of the *o*-$CoSe_2$|P and *c*-$CoSe_2$. *o*-$CoSe_2$|P sample shows discernible P peaks that indicates the existence of P dopants. The signals at 128.8/139.2 and 186.7 eV can be assigned $L_{2,3}$ and L_1 (ref. 2). The P *L*-edge is less pronounced due to the low P content in the *o*-$CoSe_2$|P structure.

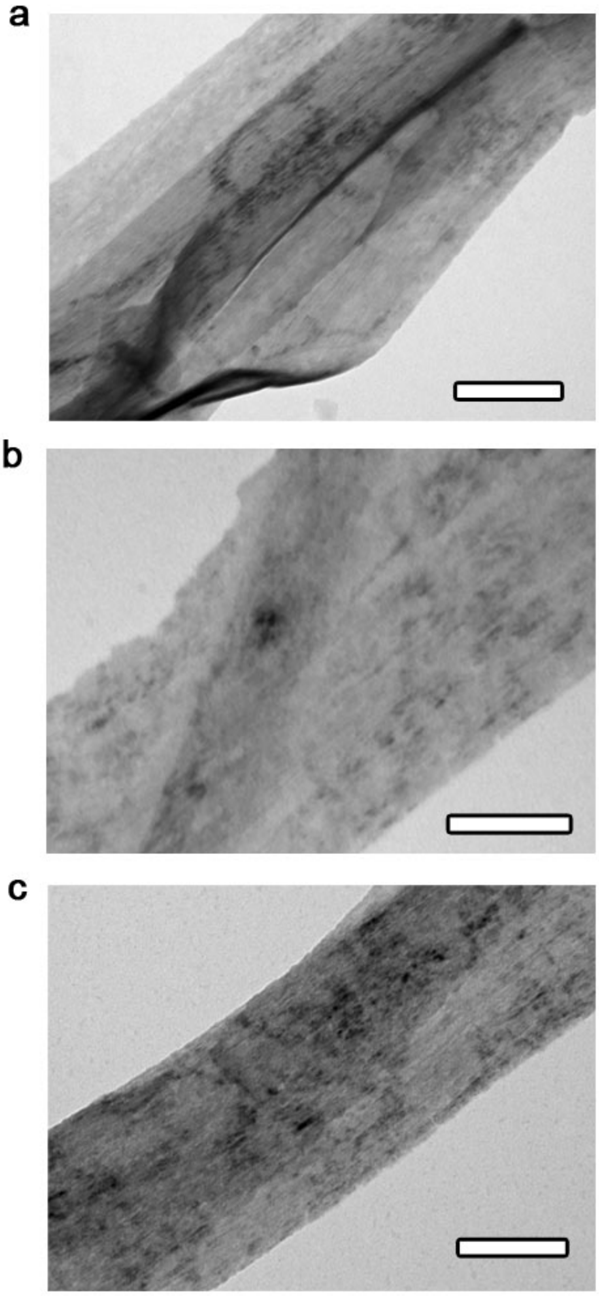

Supplementary Figure 8. Temperature-dependent experiments. TEM images of the products obtained at various temperatures: **a,** 300 ^{o}C, **b,** 350 ^{o}C and **c,** 400 ^{o}C. Scale bars, 200 nm, 100 nm and 100 nm, respectively.

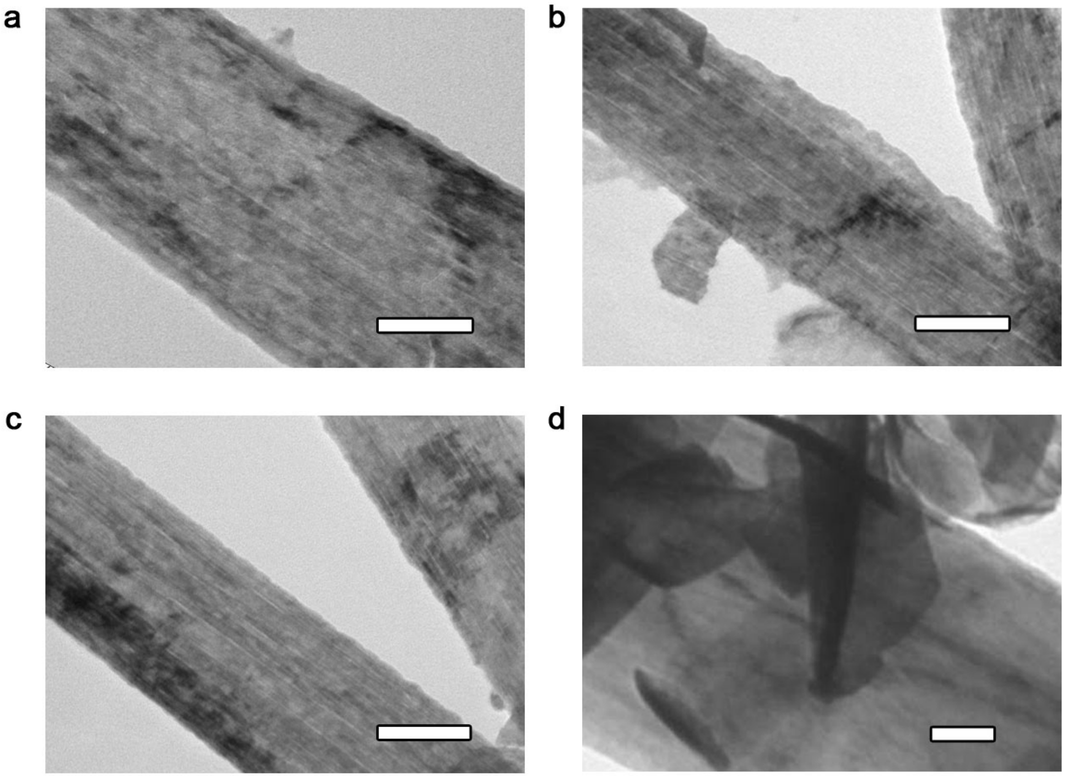

Supplementary Figure 9. Time-dependent experiments. TEM images of the products obtained at 400 ^{o}C for different times with the same precursor ratio: **a,** 1 h, **b,** 2 h, **c,** 3 h, **d,** 4 h. Scale bar, 100 nm. It shows that longer heating time leads to the disappearance of porous structure because of the Ostwald ripening process.

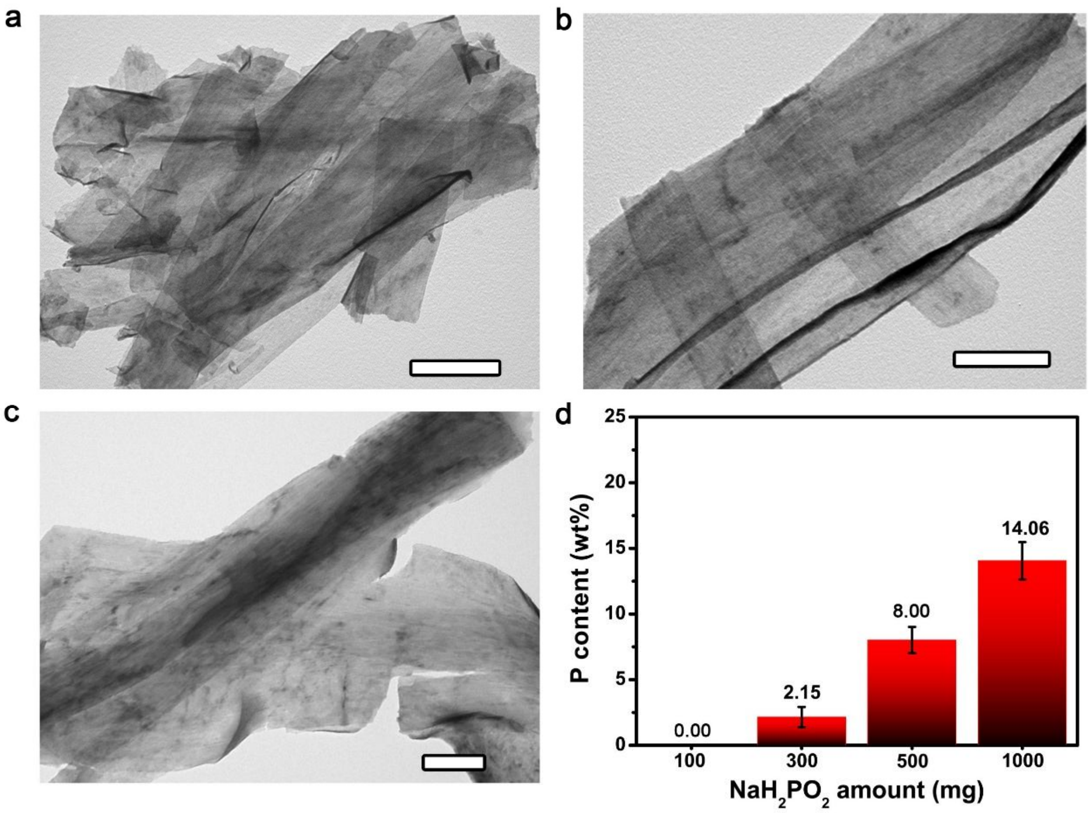

Supplementary Figure 10. Doping content effect. TEM images of the products obtained with different amount of P precursor: **a,** 100 mg; scale bar, 500 nm. **b,** 300 mg and **c,** 1 g. Scale bar, 100 nm. **d,** Diagram shows P-doping content of *o*-$CoSe_2$|P samples prepared with different amount of P precursor. It seems no significant changes in morphology of *o*-$CoSe_2$|P with various P dopants. Moreover, small amount of P precursor such as 100 mg can not generate enough PH_3 that enables the P doping process.

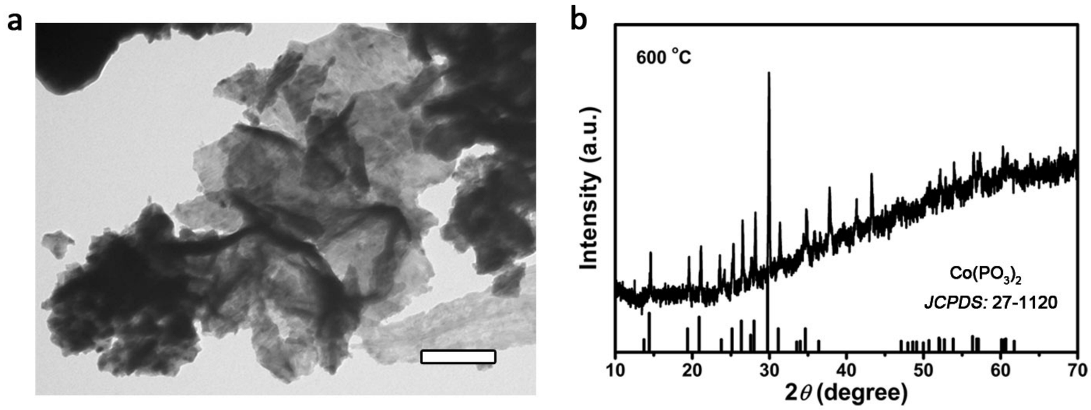

Supplementary Figure 11. Structural characterization of the product obtained at 600 °C, 30 min with 500 mg P precursor. a, TEM image of the sample. Scale bar: 200 nm. **b,** XRD pattern of the sample, which was assigned to $Co(PO_3)_2$ (JCPDS: 27-1120). It shows that very high annealed temperature can not allow for the P-doping-induced phase transition in $CoSe_2$.

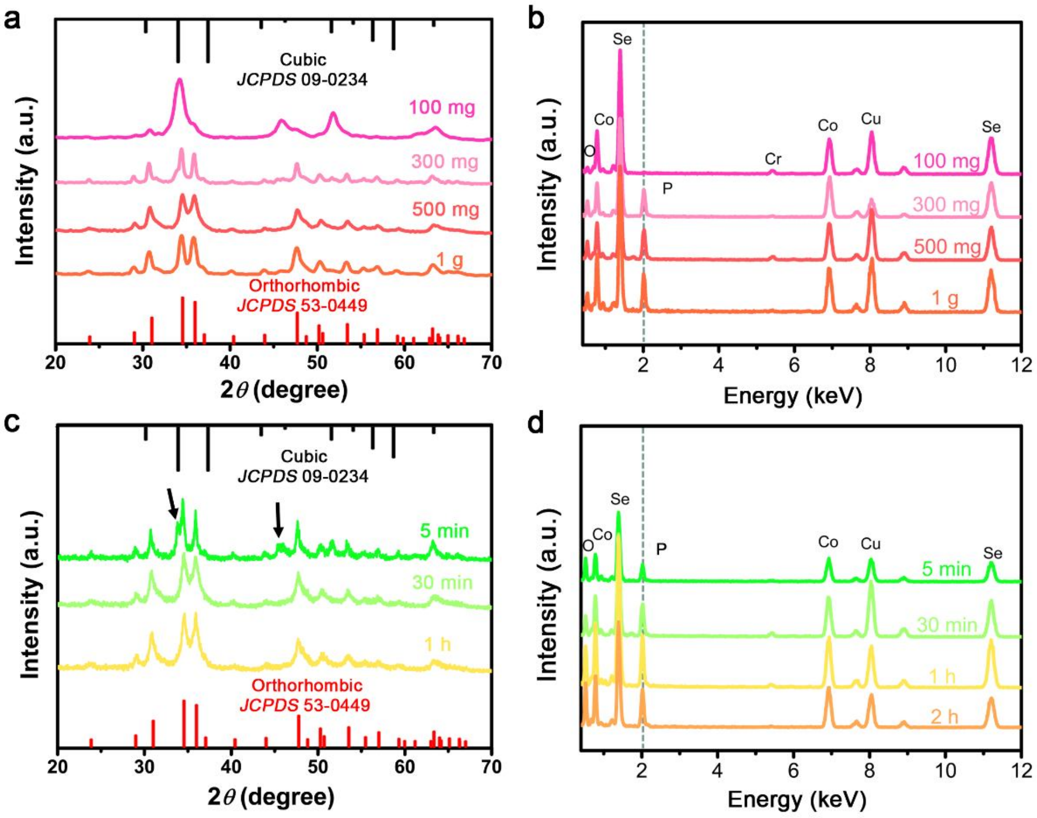

Supplementary Figure 12. XRD and EDX characterizations. a, **b,** XRD and EDX spectra of samples prepared with different amounts of P precursor, respectively. **c**, **d,** XRD and EDX spectra of samples prepared at different reaction times, respectively. It reveals that P can be doped in $CoSe_2$ structure only when the offered $NaH_2PO_2 \cdot H_2O$ is larger than 100 mg. It also reveals that the phase transition emerges at ~5 min when the temperature reaches 400 °C. The phase transition from *c*-$CoSe_2$ to *o*-$CoSe_2$|P completed at ~30 min. Cu, Cr and O signals in EDX spectra were originated from the sample holder.

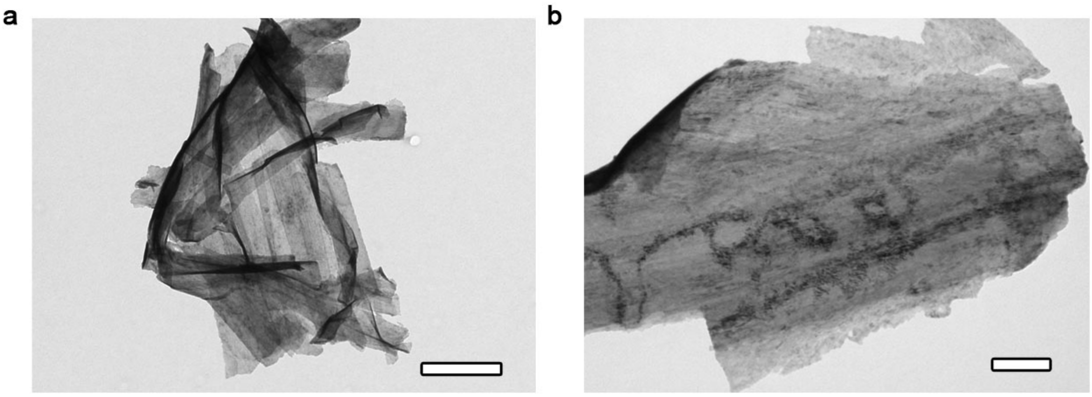

Supplementary Figure 13. TEM images of annealed *c*-$CoSe_2$ without P doping. a, b, TEM images of *c*-$CoSe_2$ that has been annealed at 400 ^{o}C under Ar flow for 30 min. Scale bars, 500 and 200 nm, respectively. It shows that the phase (see Fig. 2a in Main Text) and morphology maintain if no P precursor was added during the reaction, suggesting the important role of P that enables the structural phase transition from *c*-$CoSe_2$ to *o*-$CoSe_2$|P.

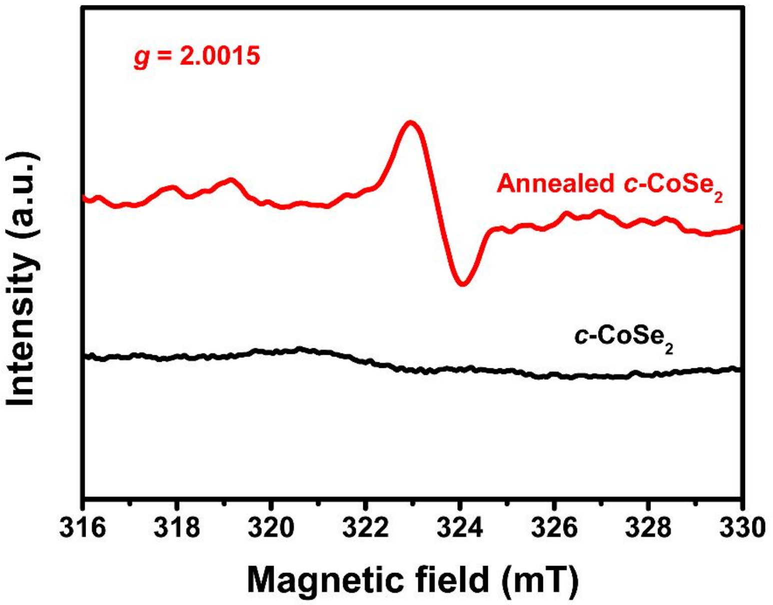

Supplementary Figure 14. EPR spectra analyses. Comparison of EPR spectra of *c*-$CoSe_2$ and annealed *c*-$CoSe_2$ without P doping at 400 ^{o}C under Ar flow. The annealed *c*-$CoSe_2$ shows a clear EPR signal with a *g* value of 2.0015, accounting for the existence of positively charged Se vacancies, consistent with a previous report[3].

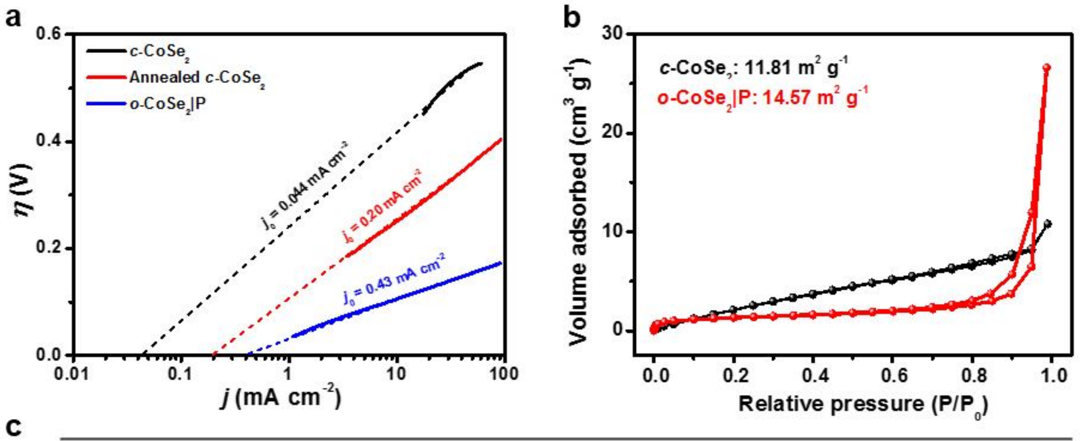

c

Catalysts	Loading (mg)	BET ($m^2\,g^{-1}$)	j_0 ($mA\ cm^{-2}$)	Normalized j_0 ($mA\ cm_{BET}^{-2}$)
c-$CoSe_2$	0.2	11.81	0.044	3.65×10^{-5}
o-$CoSe_2$|P	0.2	14.57	0.43	2.89×10^{-4}

Supplementary Figure 15. Exchange current density calculation. a, Calculated exchange current densities (j_0) for different studied catalysts by using extrapolation methods. The j_0 of 0.43 mA cm^{-2} for *o*-$CoSe_2$|P catalyst surpasses recently reported single-catalysts, and even many composite catalysts (**Supplementary Tables 1-2**), demonstrating its remarkable HER kinetics in alkali. **b,** BET surface area analysis of *c*-$CoSe_2$ and *o*-$CoSe_2$|P catalysts, respectively. **c,** Comparison of BET surface area normalized exchange current densities of *c*-$CoSe_2$ and *o*-$CoSe_2$|P, respectively. It reveals that the new *o*-$CoSe_2$|P catalyst bears the BET-normalized j_0 of 2.89×10^{-4} mA cm^{-2}, which is much larger than that of *c*-$CoSe_2$ catalyst.

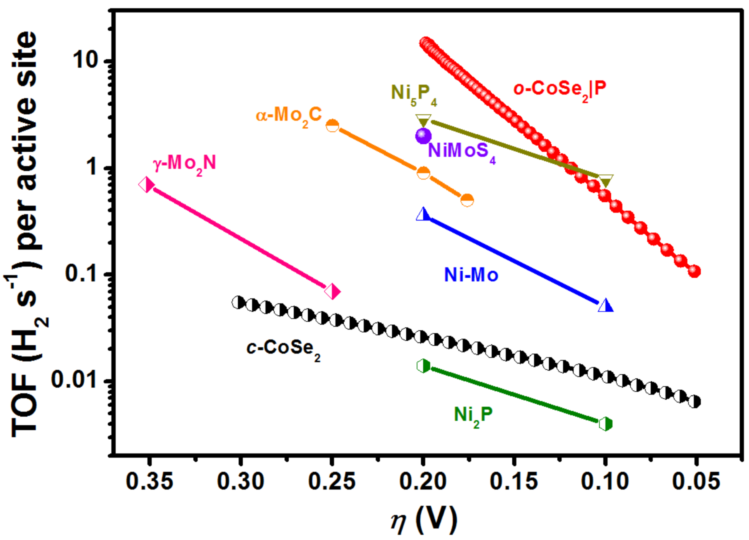

Supplementary Figure 16. Comparison of the TOF of *o*-$CoSe_2$|P with other catalysts. TOF plots of the *o*-$CoSe_2$|P catalyst together with other documented alkaline HER catalysts (**Supplementary Tables 1-2**). We determined the active site to be ~4.81×10^{16} sites per cm^2, based on the hypothesis that one Co-X_2 dumbbell offers one active site (details of the calculation are provided in **Supplementary Note 1**). In particular, *o*-$CoSe_2$|P catalyst gives TOF values of 0.65 and 14.95 H_2 s^{-1} at overpotentials of 104 (j = 10 mA cm^{-2}) and 200 mV (j = 230 mA cm^{-2}), respectively. It shows clearly that *o*-$CoSe_2$|P catalyst outperforms recently developed non-noble HER catalysts in alkali.

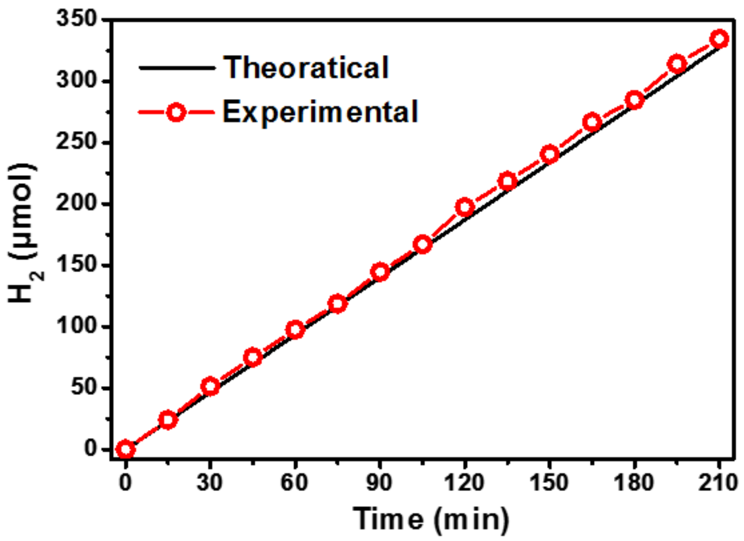

Supplementary Figure 17. Hydrogen production efficiency. Gas chromatography (GC)-measured H_2 quantity compared with theoretically calculated H_2 quantity assuming a 100% Faradic efficiency for the hydrogen evolution catalyzed by the *o*-$CoSe_2$|P catalyst in 1 M KOH at a current density of 10 mA cm^{-2}. We measured the H_2 production in a custom made two compartment cell, which is separated by a Nafion membrane. Each compartment of the cell was filled with 25 mL 1.0 M KOH solution. The headspace of the cathodic compartment was approximately 20 mL.

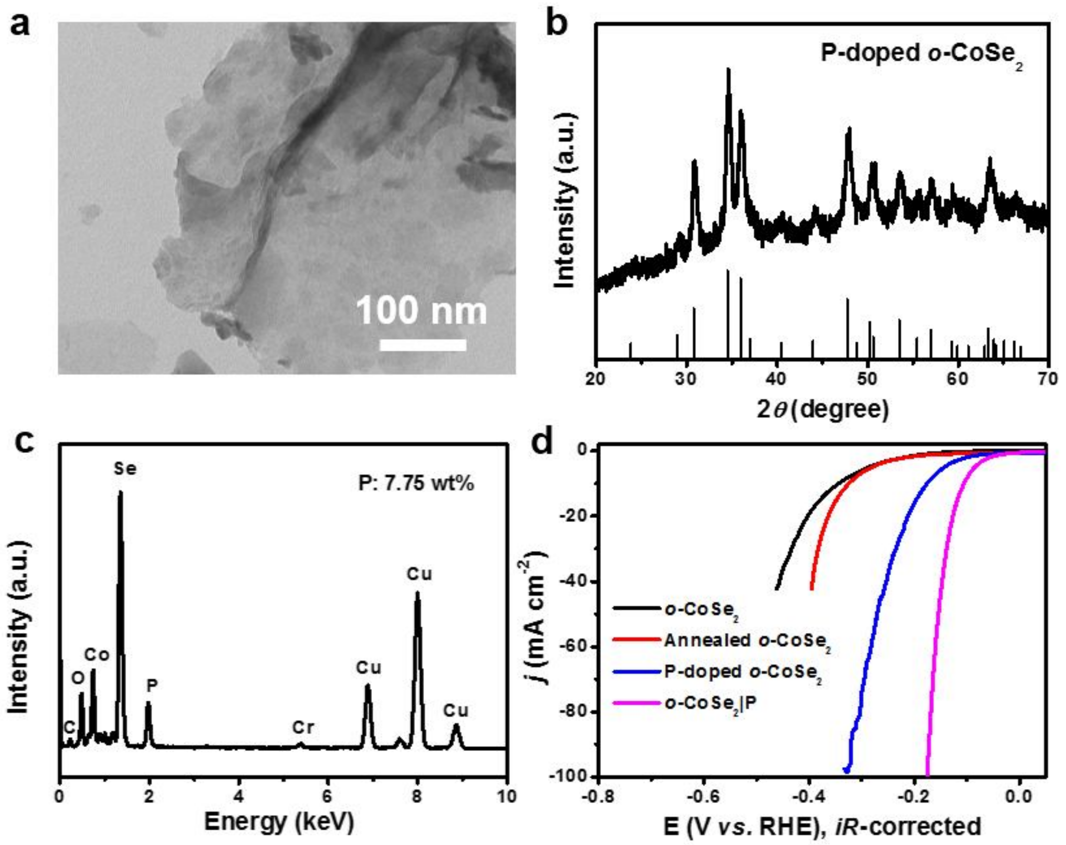

Supplementary Figure 18. Structural characterizations and HER performance of P-doped *o*-$CoSe_2$. a, TEM image, **b,** XRD pattern and **c,** EDX spectrum of the P-doped *o*-$CoSe_2$ that prepared by phosphorization of fresh *o*-$CoSe_2$ via the same method. **d,** HER polarization curves of pure *o*-$CoSe_2$, Ar-annealed *o*-$CoSe_2$, P-doped *o*-$CoSe_2$, and *o*-$CoSe_2$|P in 1 M KOH. Catalyst loading: ~1.0 mg cm^{-2}. Sweep rate: 10 mV s^{-1}. These results reveal that, although P-doped *o*-$CoSe_2$ shows good activity promotions as compared with pure *o*-$CoSe_2$, it is still inferior to the performance of the new *o*-$CoSe_2$|P catalyst. We attribute this observation to the doping-induced bonds rotation and structure reconstructions, which create additional surface sites that are HER active, further highlighting the effectiveness of this doping-inducing phase-transition method for accessing newly advanced electrocatalysts.

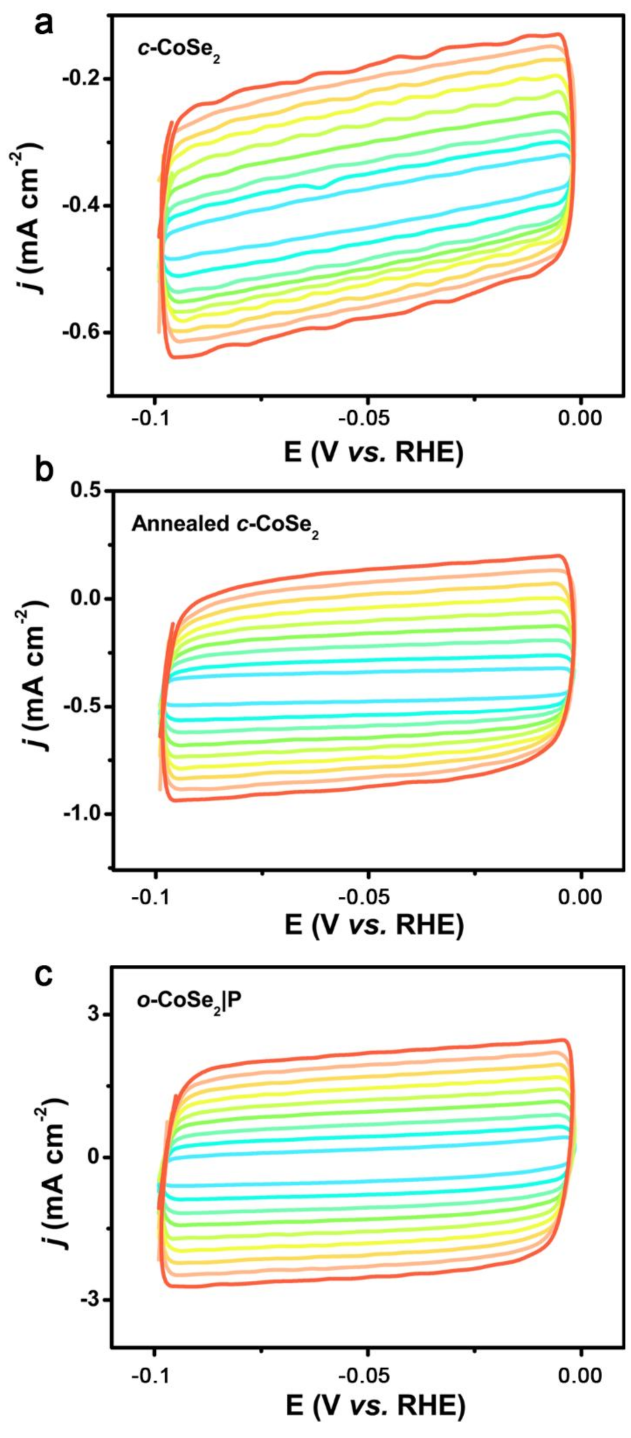

Supplementary Figure 19. Capacitance measurement. a-c, CV in the region of -0.1~0.0 V vs. RHE for various studied catalysts. The double layer capacitance (C_{dl}) is an alternative approach to estimate the effective surface area, thus offering a relative comparison. CV responses in the region of -0.1~0.0 vs. RHE could be resulted from the charging of double layer and the C_{dl} value can be estimated by plotting the Δj (j_a- j_c) at -0.05 V vs. RHE against the scan rate, where the slop is double C_{dl} (**Fig. 3e in main text**).

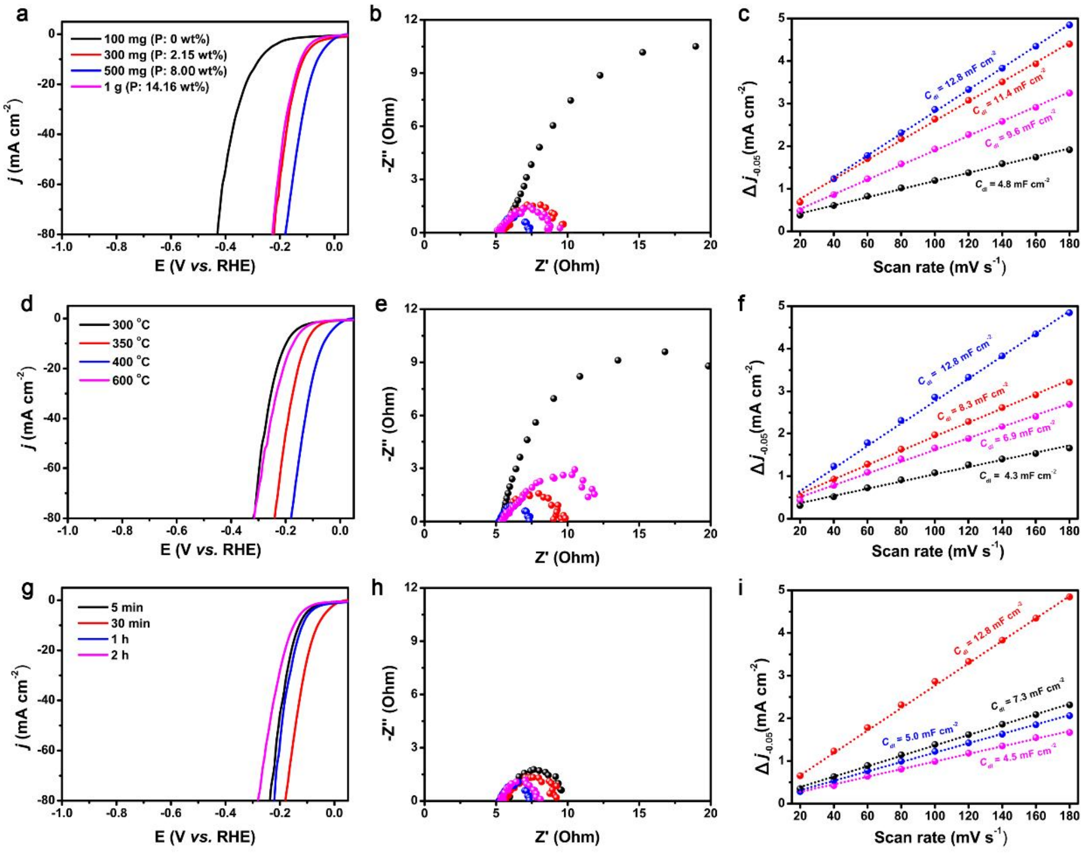

Supplementary Figure 20. Influence of synthetic parameters on the HER properties in 1 M KOH. a, **d**, **g,** HER polarization curves, and **b**, **e**, **h,** corresponding EIS Nyquist plots of *o*-$CoSe_2$|P catalysts prepared at different synthetic parameters. **c**, **f**, **i,** Plots showing the extraction of the C_{dl} for *o*-$CoSe_2$|P catalysts prepared at different synthetic parameters. It reveals that the optimized *o*-$CoSe_2$|P catalyst obtained at 400 °C for 30 min with P-doping amount of 8 wt%.

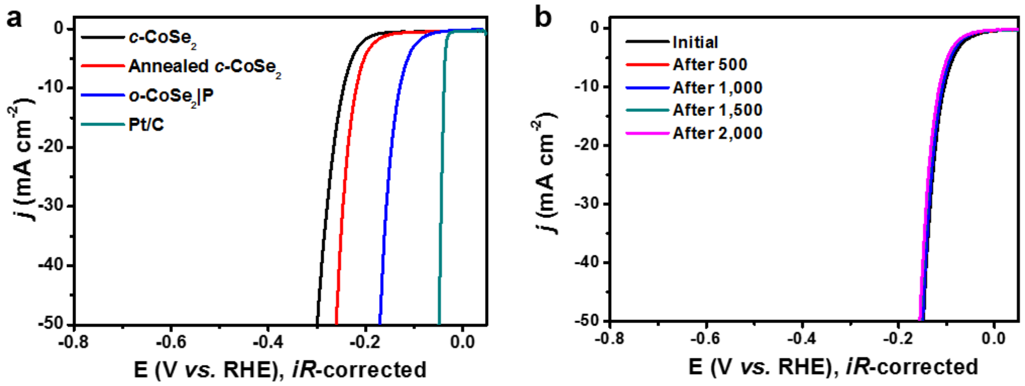

Supplementary Figure 21. HER properties in 0.5 M H_2SO_4. a, Polarization curves of *o*-$CoSe_2$|P, *c*-$CoSe_2$, annealed $CoSe_2$ and commercial Pt/C catalysts in 0.5 M H_2SO_4 (pH = 0). Catalysts loading: ~1.0 mg cm^{-2}. Sweep rate: 10 mV s^{-1}. **b,** Polarization curves recorded from *o*-$CoSe_2$|P catalyst before and after 2,000 CV cycles.

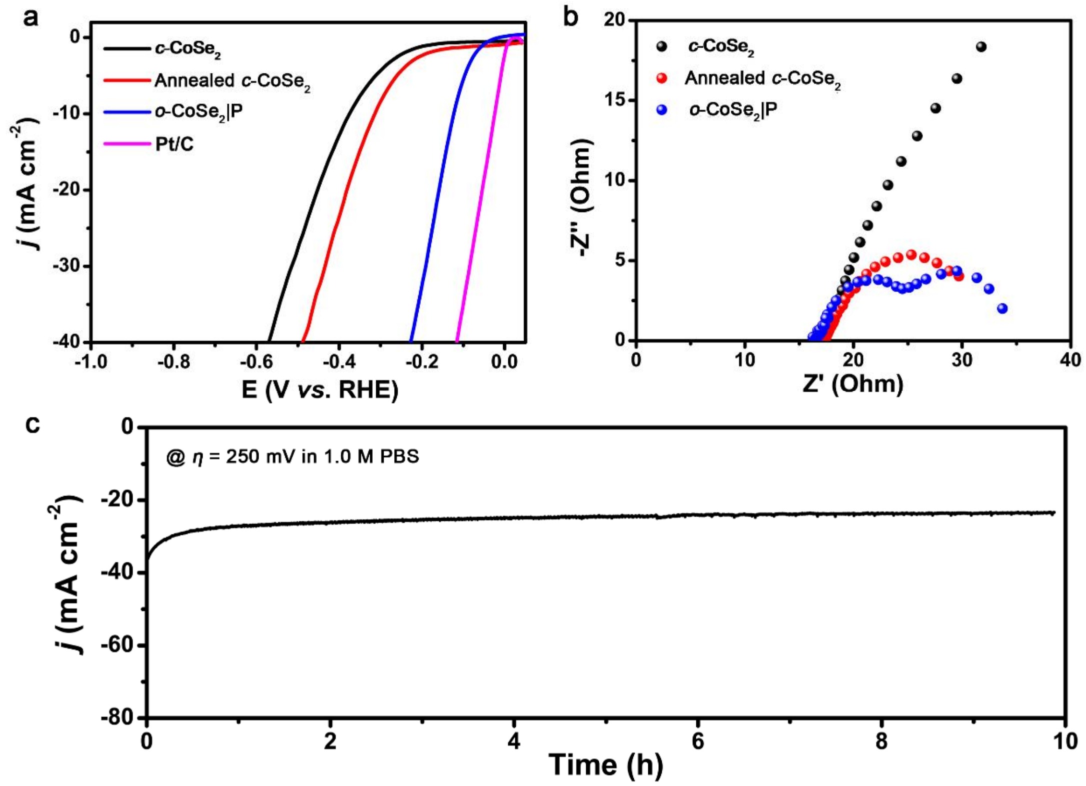

Supplementary Figure 22. HER properties in PBS solution (pH 7.02). a, HER polarization curves of *o*-$CoSe_2$|P, *c*-$CoSe_2$, annealed *c*-$CoSe_2$ and commercial Pt/C catalysts. Catalyst loading: ~1.0 mg cm^{-2}. Sweep rate: 10 mV s^{-1}. **b,** EIS Nyquist plots of *o*-$CoSe_2$|P, *c*-$CoSe_2$, and annealed *c*-$CoSe_2$. **c,** Chronoamperometric responses (*j* ~ *t*) recorded on *o*-$CoSe_2$|P at a constant overpotential of 250 mV in 1 M PBS.

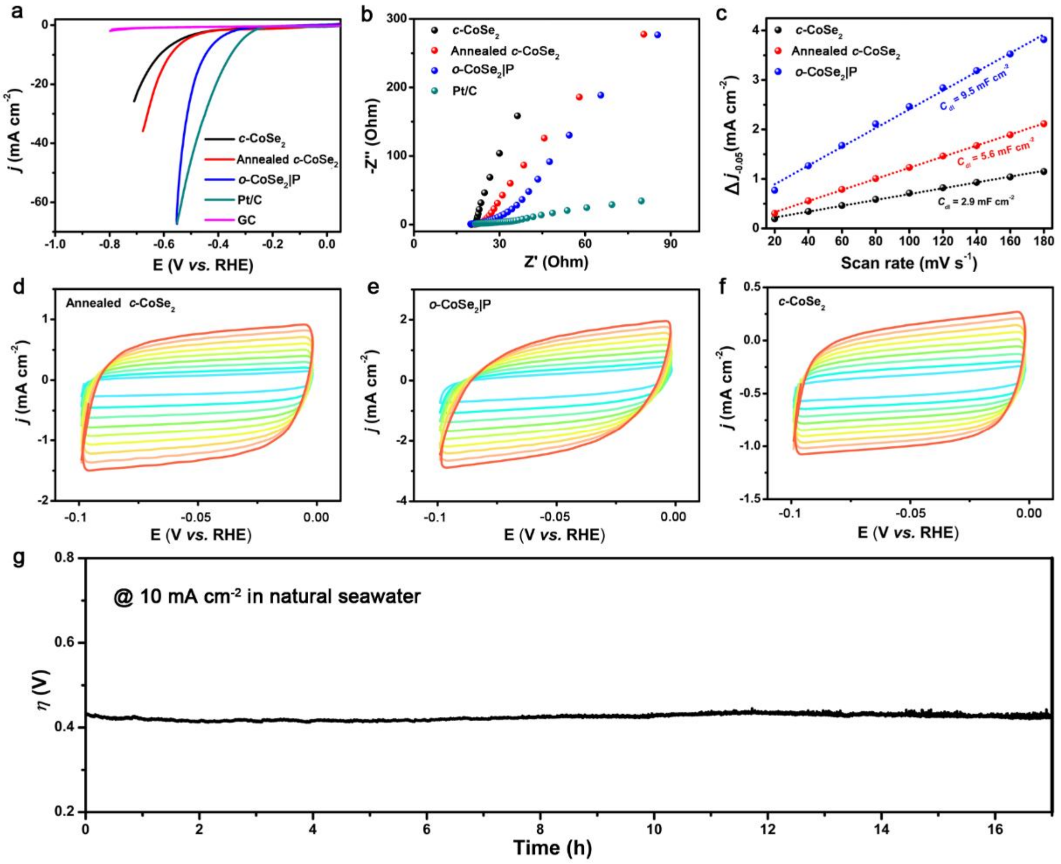

Supplementary Figure 23. HER properties in seawater (Gulf Stream in the Gulf of Mexico. pH 7.84). **a,** HER polarization curves of *o*-$CoSe_2$|P, *c*-$CoSe_2$, annealed $CoSe_2$ and commercial Pt/C catalysts. Catalysts loading: ~1.0 mg cm^{-2}. Sweep rate: 10 mV s^{-1}. **b,** EIS Nyquist plots of *o*-$CoSe_2$|P, *c*-$CoSe_2$, and annealed *c*-$CoSe_2$. **c,** Plot showing the extraction of the C_{dl} for the different studied catalysts. **d-f,** CV in the region of -0.1 ~ 0.0 V vs. RHE for different studied catalysts. **g,** Chronoamperometric responses ($\eta \sim t$) recorded on *o*-$CoSe_2$|P at a constant current density of 10 mA cm^{-2} in seawater.

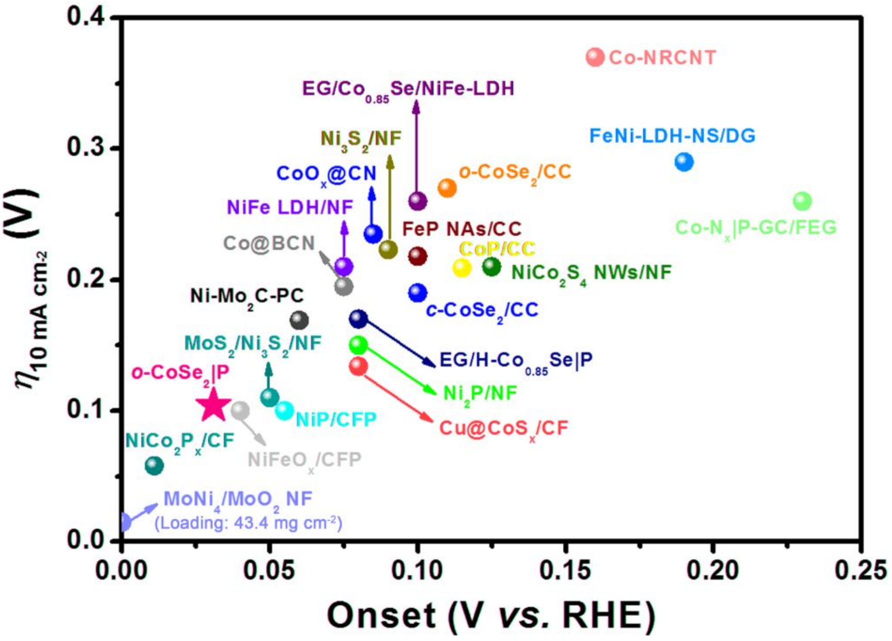

Supplementary Figure 24. Comparison of the HER property of *o*-$CoSe_2$|P with other reported composite catalysts in alkaline solutions. η_{onset} versus $\eta_{10\ mA/cm^2}$ electrocatalytic property map for *o*-$CoSe_2$|P with various reported composite HER electrocatalysts in alkali (also see **Supplementary Table 2**). We note that some catalysts take very high catalyst loadings for comparison. For example, $MoNi_4/MoO_2$ NF catalyst with a very high loading of 43.4 mg cm^{-2}.

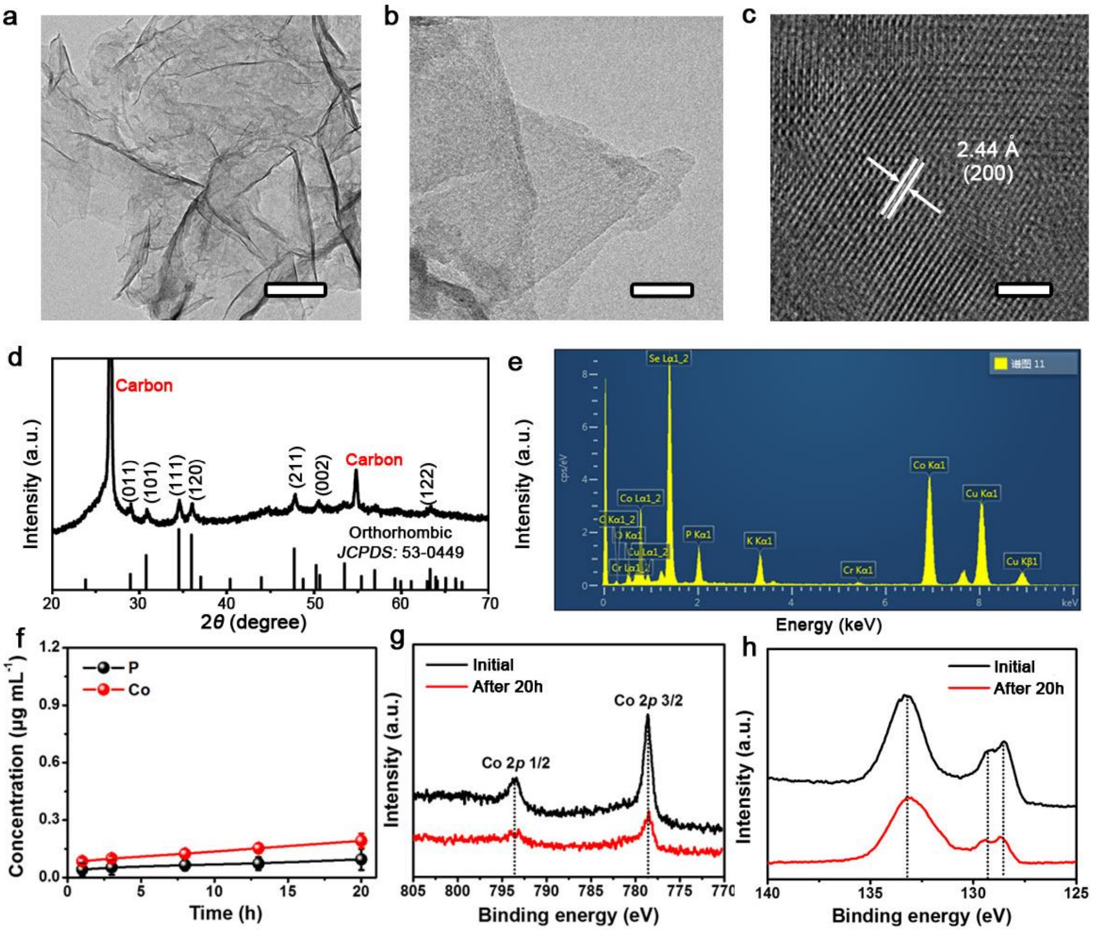

Supplementary Figure 25. Stability study of *o*-$CoSe_2$|P catalyst. a, **b,** TEM images with different magnification (Scale bar, 100 amd 20 nm, respectively) and **c,** HRTEM image (scale bar, 2 nm) of *o*-$CoSe_2$|P after a 20 h chronoamperometry measurement at a constant 250 mV overpotential. **d,** XRD pattern of *o*-$CoSe_2$|P-modified carbon fiber paper electrode after the stability test. **e,** EDX spectrum of *o*-$CoSe_2$|P after the stability test. **f,** ICP results show the concentration of P and Co in the electrolyte at different time intervals during the HER stability test. Only trace amount of Co and P were detected, which might be attributed to the catalyst fell off from the carbon paper substrate. The error bars represent the s.d. of three replicate measurements. **g,** Co 2*p*, and **h,** P 2*p* XPS spectra of the *o*-$CoSe_2$|P catalyst before and after stability test. These results demonstrate the remarkable catalytic robustness of *o*-$CoSe_2$|P catalyst in alkaline electrolyte.

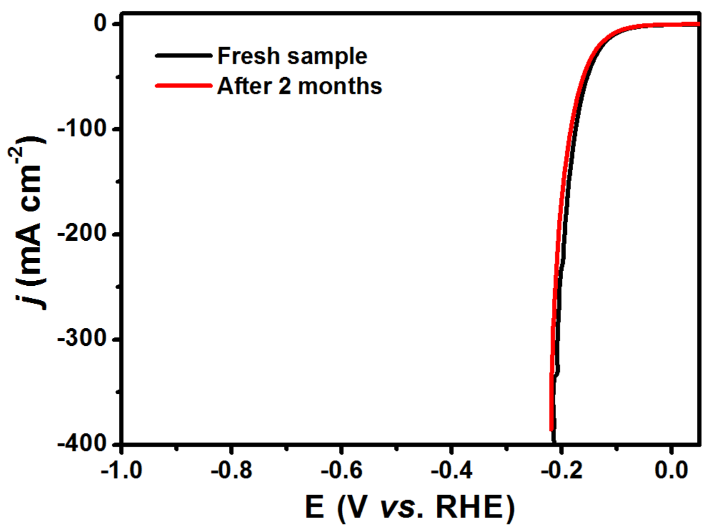

Supplementary Figure 26. Storage stability. Polarization curves (with *iR*-corrected) recorded from *o*-$CoSe_2$|P before and after 2 months of storage under lab environment (stored in an airtight sample tube), revealing that no obvious degradation of HER activity was detected and thereby demonstrating the good storage stability.

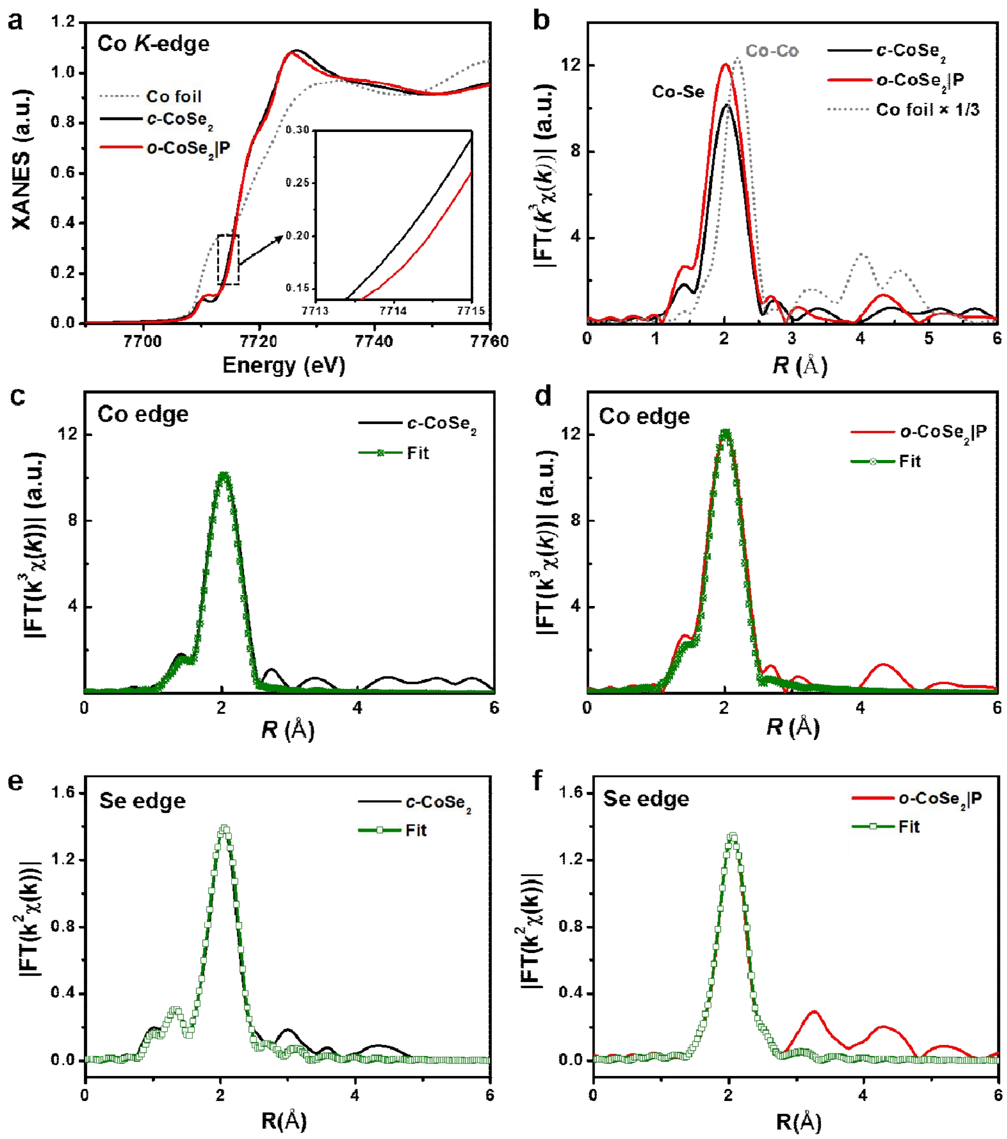

Supplementary Figure 27. XAFS spectra of *c*-$CoSe_2$ and *o*-$CoSe_2$|P. a, Co *K*-edge XANES spectra. The Inset shows the enlarged spectra at the Co *K*-edge. The edge position of *o*-$CoSe_2$|P shifted to higher energy indicates that the electron-deficient on Co sites. **b,** EXAFS Fourier transformed k^3-weighted $\chi(k)$ function spectra of *c*-$CoSe_2$, *o*-$CoSe_2$|P and Co foil. **c, d,** The EXAFS fitting results of the Co edge for *c*-$CoSe_2$ and *o*-$CoSe_2$|P, respectively. The peak at 2.04 Å of *c*-$CoSe_2$, corresponding to Co-Se scattering paths. In contrast, for the *o*-$CoSe_2$|P, the peak intensity is increased and shifts to lower *R* by 0.02 Å. The increased peak intensity can be attributed to the modified surface structural disorder of *o*-$CoSe_2$|P after thermal treatment. The low-*R* shift of Co-Se peak is due to the partial replacement of Se atoms by smaller P.**e, f,** The EXAFS fitting results of the Se edge for *c*-$CoSe_2$ and *o*-$CoSe_2$|P, respectively. Quantitative structural fitting parameters were given in **Supplementary Table 3**.

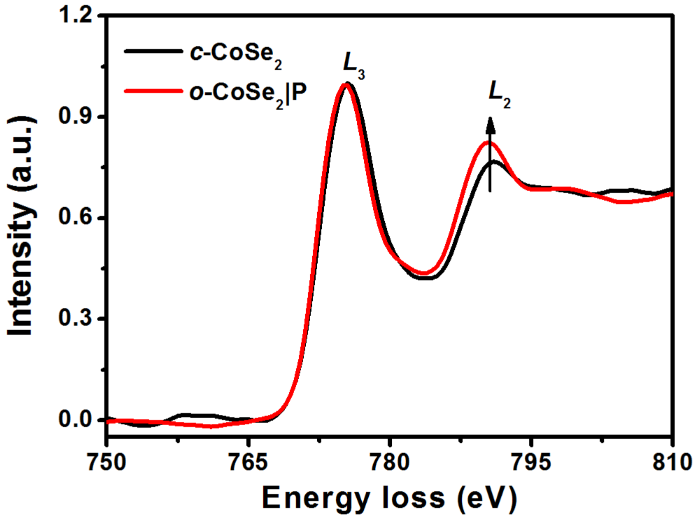

Supplementary Figure 28. Co *L*-edge EELS spectra of *c*-$CoSe_2$ and *o*-$CoSe_2$|P. Background-subtracted EELS spectra of the Co $L_{2,3}$ edges obtained for the *c*-$CoSe_2$ and *o*-$CoSe_2$|P, which could reveal the excitation of the Co $2p_{1/2}$ or Co $2p_{3/2}$ electrons from inner shell into the first available unoccupied states above the Fermi-level. And the L_2/L_3 ratio would be increased when the *d*-band occupancy decreases, which means the increased oxidation state of the atoms[4]. Clearly, the *o*-$CoSe_2$|P has a increased intensity of L_2 edge, suggesting the formal oxidation of Co due to the electron transfer from Co to P.

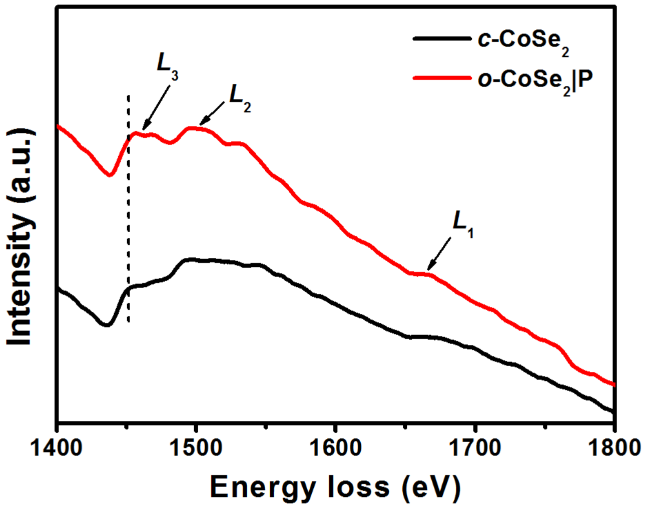

Supplementary Figure 29. Se *L*-edge EELS spectra of $CoSe_2$ and *o*-$CoSe_2$|P. The large background below the Se *L*-edge arises from the inelastic scattering events at lower energies, such as generation of secondary electrons, Plasmon losses and ionization of the Se M shell[5]. EELS shift to higher energy indicates that the *o*-$CoSe_2$|P with higher oxidation states because of the electron transfer from Se to P.

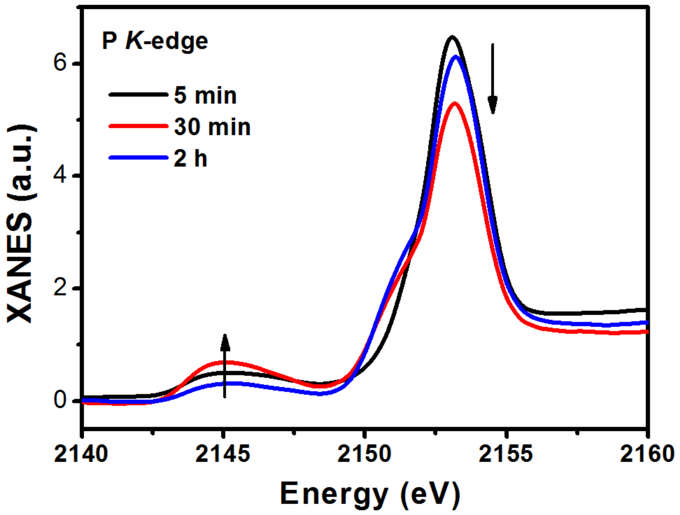

Supplementary Figure 30. P *K*-edge XANES of *o*-$CoSe_2$|P prepared at different annealing times. The samples exhibit two major features that were centered at 2145.0 and 2153.1 eV. The feature at 2145.0 eV is attributed to P in a phosphide form, consistent with the presence of Co-P (ref. 6). It suggests that the optimized *o*-$CoSe_2$|P sample has more Co-P bonds than others. The intense white line centred at 2153.1 eV is assigned to a transition of the P 1*s* electron into an unoccupied valence electronic state[7]. The depressed intensity and the shifted pre-edge of optimized *o*-$CoSe_2$|P are attributed to the electrons transfer form neighboring Co or Se atoms to P.

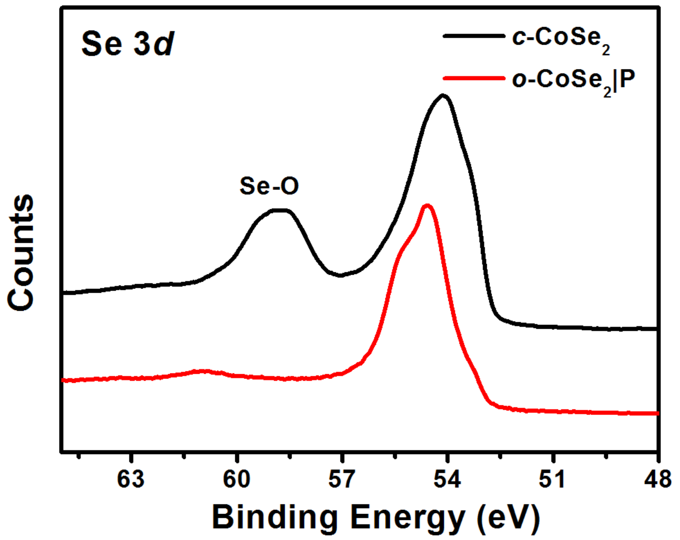

Supplementary Figure 31. High-resolution XPS spectra. High-resolution Se 3*d* XPS spectra for *c*-$CoSe_2$ and *o*-$CoSe_2$|P. The disappeared oxidation state feature of the Se species for *o*-$CoSe_2$|P catalyst indicates that the participation of P can mitigate the surface oxidation process.

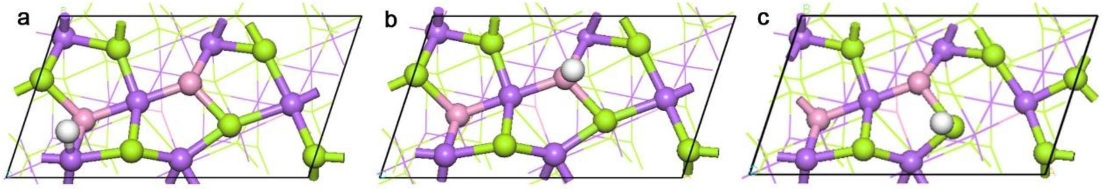

Supplementary Figure 32. H adsorption on different atom sites. a-c, The adsorption sites of H atom on top of Co, P and Se atoms on the (111) facet of *o*-$CoSe_2$|P. The *o*-$CoSe_2$|P surface was built by replacing Se atoms with P in the *o*-$CoSe_2$ (111) and the ratio of P atoms is ~8%. Different P distributed structures have been tested, and the most stable surface was used to calculate the hydrogen adsorption. Based on the calculation, replacing Se by P atoms is able to switch the pristine Co-Se and Se-Se bonds to shorter Co-P and Se-P bonds. The altered structures are able to modulate the surface states and charge distribution, thus optimizing the Gibbs free-energy (ΔG_{H*}). The corresponding ΔG_{H*} are shown in Table S4.

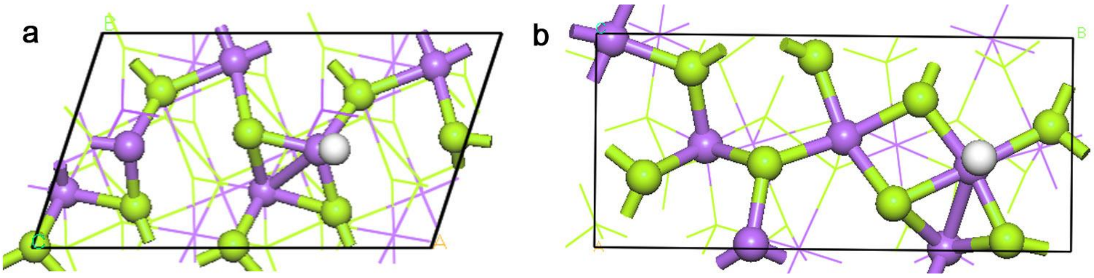

Supplementary Figure 33. H adsorption on selected facets of studied catalysts. a, b, The adsorption site of H atom on the *o*-$CoSe_2$ (111) and *c*-$CoSe_2$ (210) facets, respectively. The calculated lattice parameters of orthorhombic and cubic $CoSe_2$ are a=3.636 Å, b=4.894 Å, c=5.875 Å and a=b=c=5.860Å, respectively, in accordance with previous experimental and theoretical studies[8,9]. (2×1) orthorhombic $CoSe_2$ (111) and cubic $CoSe_2$ (210) surfaces are adopted in the calculations, both with Co-terminated surfaces. 9 and 11 layers (here the distance between the neighboring layer is larger than 0.9 Å) in the orthorhombic $CoSe_2$ (111) and cubic $CoSe_2$ (210) surfaces, respectively, both with the bottom three layers fixed.

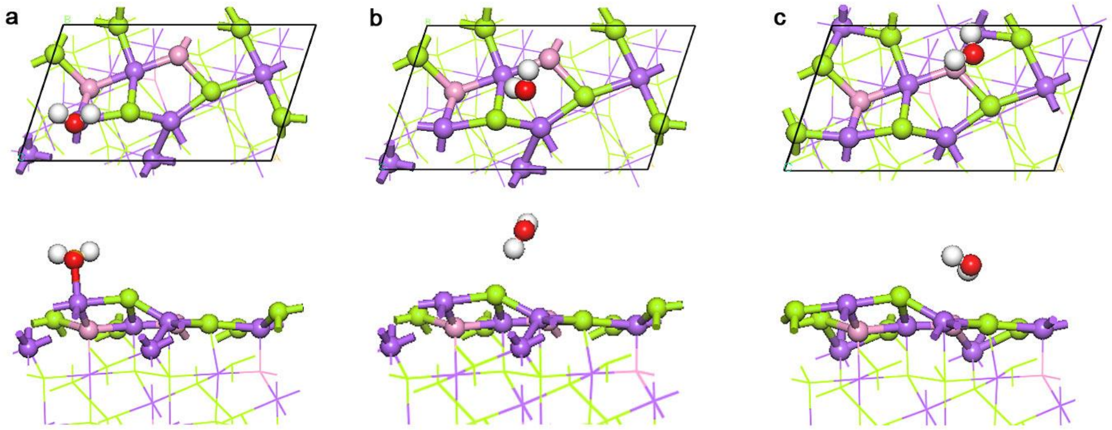

Supplementary Figure 34. H_2O adsorption. a-c, The adsorption site of H_2O molecule on the top of Co, P and Se atoms in the optimized (111) facet of *o*-$CoSe_2$|P, respectively.

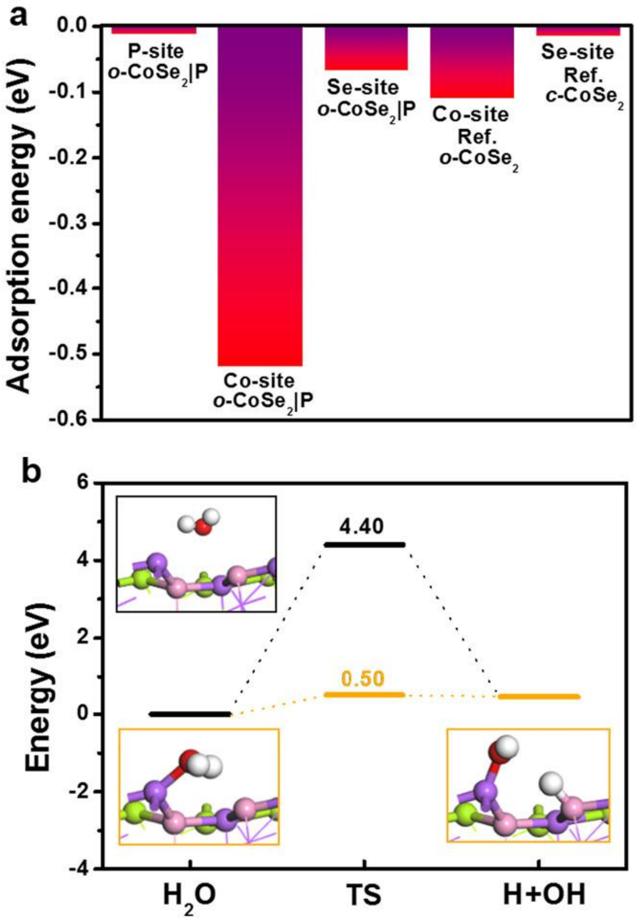

Supplementary Figure 35. Adsorption and dissociation of H_2O molecule on *o*-$CoSe_2$|P surface. a, Adsorption energy of H_2O molecule. Comparison of the adsorption energy of H_2O on different atoms on the (111) facet of *o*-$CoSe_2$|P. The data of *c*-$CoSe_2$ and *o*-$CoSe_2$ are derived from ref. 10. It shows that H_2O molecule on the Co atom sites of *o*-$CoSe_2$|P exhibits the lowest water adsorption energy of -0.52 eV as compared to that of P sites (-0.068 eV) and Se sites (-0.0127 eV), which suggests that the initial H_2O molecules can readily adsorbed on the Co sites of *o*-$CoSe_2$|P. **b,** H_2O dissociation in vacuum and on *o*-$CoSe_2$|P surface. Because the weak adsorption energy of H_2O molecular on Se and P sites, there is almost no H_2O molecular on these sites, thus the dissociated energy of H_2O on these sites are similar in vacuum. As to Co sites, the energy barrier of H_2O dissociation could be largely reduced from 4.40 eV (in vacuum) to 0.50 eV, suggesting that Co sites not only bear a strong water affinity, but also easily activate H_2O to generate intermediate H and OH species. Hence, these results evidence the synergistic interplay between Co (water adsorption and dissociation) and P (water reduction) that gives rise to the remarkable HER energetics.

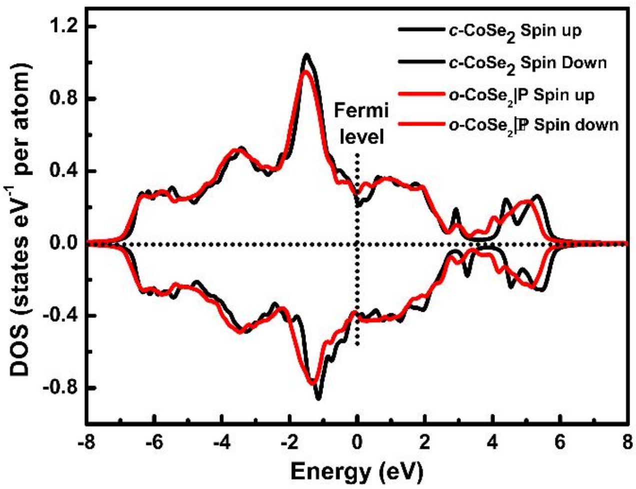

Supplementary Figure 36. DOS diagram. Comparison of the total DOS diagram for *c*-$CoSe_2$ and *o*-$CoSe_2$|P catalysts.

Supplementary Table 1: Comparison of catalytic parameters of *o*-$CoSe_2$|P and other non-noble-metal HER single-catalysts in alkaline media.

Catalyst	Loading (mg cm^{-2})	Electrolyte	Onset potential @ 1 mA cm^{-2} (mV *vs.* RHE)	$\eta_{10\ mA\ cm^{-2}}$ (mV)	Tafel slope (mV dec^{-1})	j_0 (mA cm^{-2})	TOF (H_2 s^{-1} per site)	Ref.
CoMoS	0.84	1 M KOH	42	98	82	0.74	N/A	11
$Co(S_{0.71}Se_{0.29})_2$	~1	1 M KOH	*ca.* 70	145	85.7	N/A	N/A	12
O-Co_2P	0.2	1 M KOH	110	160	61.1	N/A	N/A	13
N,S-graphitic sheets	0.643	0.1 M KOH	230	310	112	N/A	N/A	14
$Pr_{0.5}$BSCF	0.232	1 M KOH	179	237	45	N/A	N/A	15
$NiCo_2O_4$	~1	1 M NaOH	50	110	49.7	N/A	N/A	16
$NiMo_3S_4$	0.3	0.1 M KOH	59	257	98	0.039	N/A	17
VOOH	0.8	1 M KOH	100	164	104	N/A	N/A	18
MoB	0.9	1 M KOH	140	220	59	2.0×10^{-3}	N/A	19
Mo_2C	1.1	1 M KOH	140	170	54	3.8×10^{-3}	N/A	19
Ni_2P	0.14	1 M KOH	120	220	N/A	N/A	N/A	20
MoC_x	0.8	1 M KOH	80	151	59	0.029	N/A	21
Ni-Co-P	0.286	1 M KOH	133	150	60.6	N/A	N/A	22
$Ni_{0.75}Ag_{0.25}$	N/A	0.1 M KOH	110	300	N/A	N/A	N/A	23
MoP	0.86	1 M KOH	75	128	48	4.6×10^{-2}	N/A	24
$NiSe_2$ NCs	~1	1 M KOH	310	544	139	5.0×10^{-3}	N/A	25
c-$CoSe_2$ NCs	~1	1 M KOH	300	520	126	8.6×10^{-3}	N/A	25
α-Mo_2C	0.102	1 M KOH	100	176	58	N/A	$0.9_{\ 200\ mV}$	26
γ-Mo_2N	0.102	1 M KOH	240	353	158	N/A	$0.7_{\ 200\ mV}$	26
$Ni_{0.90}Fe_{0.10}PS_3$	0.51	1 M KOH	N/A	72	73	N/A	N/A	27
o-$CoSe_2$|P	1.02	1 M KOH	31	104	69	0.43	$14.95_{\ 200mV}$	This work

$Pr_{0.5}$BSCF = $Pr_{0.5}(Ba_{0.5}Sr_{0.5})_{0.5}Co_{0.8}Fe_{0.2}O_{3-\delta}$; NCs = Nanocrystals; NBs = Nanobelts

Supplementary Table 2: Comparison of catalytic parameters of *o*-$CoSe_2$|P and other non-noble-metal HER composite-catalysts in alkaline media.

Catalyst	Loading (mg cm^{-2})	Electrolyte	Onset potential @ 1 mA cm^{-2} (mV *vs.* RHE)	$\eta_{10\ mA\ cm^{-2}}$ (mV)	Tafel slope (mV dec^{-1})	j_0 (mA cm^{-2})	TOF (H_2 s^{-1} per site)	Ref.
CoP/CC	0.92	1 M KOH	115	209	129	N/A	N/A	28
NiP/CFP	25.8	1 M KOH	55	100	85.4	N/A	N/A	29
Ni_2P/NF	N/A	1 M KOH	80	150	86	N/A	N/A	30
c-$CoSe_2$/CC	N/A	1 M KOH	100	190	85	N/A	N/A	31
o-$CoSe_2$/CC	N/A	1 M KOH	110	270	120	N/A	N/A	31
FeP NAs/CC	1.5	1 M KOH	100	218	146	N/A	N/A	32
CoP-MNA/CFP	N/A	1 M KOH	N/A	54	51	0.857	N/A	33
$NiCo_2P_x$/CF	N/A	1 M KOH	11	58	34.3	N/A	0.056 $_{100mV}$	34
EG/H-$Co_{0.85}$Se|P	2.1	1 M KOH	*ca.* 80	170	86	N/A	N/A	35
EG/$Co_{0.85}$Se/NiFe-LDH	4.0	1 M KOH	100	260	160	0.22	N/A	36
$Ni_{0.89}Co_{0.11}Se_2$ MNSN/NF	2.62	1 M KOH	N/A	85	52	N/A	N/A	37
$NiMoS_4$/Ti mesh	1.71	1 M KOH	N/A	138	97	N/A	0.725 $_{148\ mV}$	38
Ni_3S_2/NF	1.6	1 M KOH	90	223	N/A	N/A	N/A	39
$NiCo_2S_4$ NWs/NF	N/A	1 M KOH	125	210	58.9	N/A	N/A	40
MoS_2/Ni_3S_2/NF	9.7	1 M KOH	50	110	83.1	N/A	N/A	41
Cu@CoS_x/CF	~3.9	1 M KOH	*ca.* 80	134	N/A	N/A	N/A	42
Ni_5P_4 pellet	177	1 M KOH	N/A	49	98	N/A	2.9 $_{200\ mV}$	43
Ni_2P pellet	177	1 M KOH	N/A	69	118	N/A	0.014 $_{200\ mV}$	43
NiFe LDH-NS/DG	2	1 M KOH	190	290	110	N/A	N/A	44
NiFe LDH/NF	N/A	1 M NaOH	75	210	58.9	N/A	N/A	45
CoO_x@CN	0.42	1 M KOH	85	235	115	N/A	N/A	46
Mn_3O_4 MTA/NF	N/A	1 M KOH	100	190 (20 mA cm^{-2})	98	N/A	N/A	47
Ni/NiO-CNT	0.28	1 M KOH	N/A	80	82	N/A	N/A	48
$NiFeO_x$/CFP	~1.6	1 M KOH	40	100	84.6	N/A	N/A	49
Co@BCN	N/A	1 M KOH	75	195	67.3	0.013	N/A	50
Co-NRCNT	0.28	1 M KOH	160	370	N/A	N/A	N/A	51
Co-N_x|P-GC/FEG	0.12	1 M KOH	230	260	N/A	0.05	N/A	52
GO-PANi31-FP	0.5	0.1 M KOH	*ca.* 400	520	N/A	N/A	N/A	53
Ni-Mo_2C-PC	0.5	1 M KOH	60	169	101	0.2	N/A	54
Ni-Mo nanopowder	1	1 M NaOH	N/A	~ 80	N/A	N/A	N/A	55
$MoNi_4$/MoO_2 NF	43.4	1 M KOH	~0	15	30	1.24	N/A	56
o-$CoSe_2$|P	1.02	1 M KOH	31	104	69	0.43	14.95 $_{200mV}$	This work

CC = Carbon cloth; CFP = Carbon fiber paper; NF = Ni foam; NAs = Nanowire arrays; CF = Carbon felt; EG = Exfoliated graphene; LDH = Layered double hydroxide; MNSN = Mesoporous nanosheet networks; NWs = Nanowires; DG = Defective graphene; NS= Nanosheet; CN = N-doped carbon; MTA = Microtube arrays; CNT = Carbon nanotube; BCN = N, B codoped carbon cages; NRCNT = N-riched CNT; GC = Carbon electrode; FEG = Flexible EG; PANi = polyaniline; FP = N, P, F-doped; PC = Porous carbon.

Supplementary Table 3: EXAFS data fitting results for the studied catalysts.

Sample	Shell	N	R_j (Å)	σ^2 ($\times10^{-3}$Å^2)	E_0 (eV)
c-$CoSe_2$	Co-Se	6±0.4	2.42±0.2	6.0	2.3
o-$CoSe_2$|P	Co-Se	5.2±0.4	2.42±0.2	5.0	1.2
	Co-P	0.8±0.4	2.38±0.2	5.2	1.2
c-$CoSe_2$	Se-Co	3.1±0.1	2.43±0.1	5.8	-3
	Se-Se	1.1±0.1	2.44±0.1	4.8	-4
o-$CoSe_2$|P	Se-Co	2.8±0.2	2.39±0.2	3.5	6
	Se-Se	0.9±0.1	2.50±0.2	4.3	6.8
	Se-P	0.2±0.1	2.41±0.1	5.7	5.2

N, coordination number; R, distance between centre and backscatter atoms; σ^2, the Debye-Waller factor value; ΔE^0 (eV), inner potential correction to account for the difference in inner potential between the sample and the reference compound. S_0 fitting from Co, Se foil defined as 0.68. Because of the low P-doping content in the *o*-$CoSe_2$|P structure, the Co-P or Se-P scattering paths could not be well fitted. It is also unlikely to fit the Se-Se scattering path, owing to the similar R values of Co-Se and Se-Se.

Supplementary Table 4: The ΔE_{ads}, $E_{ZPE}(H^*)$, ΔE_{ZPE} and $\Delta G(H^*)$ values of the H^* adsorbed on the various catalysts surfaces.

**Models**	**Adsorption site**	**ΔE_{ads} (eV)**	**$E_{ZPE}(H^*)$ (eV)**	**ΔE_{ZPE} (eV)**	**$\Delta G(H^*)$ (eV)**
o-$CoSe_2$|P	P-site	-0.36	0.22	0.078	-0.08
	Se-site	0.88	0.195	0.053	1.14
	Co-site	-0.03	0.176	0.034	0.21

Supplementary Note 1: Calculation of the active site density and TOF. To calculate the active surface site density and per-site TOF for *o*-$CoSe_2$|P catalyst, we adopt the method applied by Jin *et al.*[57]. We used CV to measure the C_{dl} and further calculated the active surface area of *o*-$CoSe_2$|P on GC electrode. The polished GC with a $C_{dl} \approx 0.1$ mF cm^{-2} was used as a control. The roughness factors (R_f) of *o*-$CoSe_2$|P and *c*-$CoSe_2$ can be calculated by using the following formular:

$$R_f(o - \mathrm{CoSe_2|P}) = \frac{12.8\ \mathrm{mF\ cm^{-2}}}{0.1\ \mathrm{mF\ cm^{-2}}} = 128 \quad (1)$$

The number of surface catalytic sites on the surface of flat *o*-$CoSe_2$|P catalys can be calculated based on the crystal structure of orthorhombic $CoSe_2$|P, using its lattic parameter (a=4.85 Å, b=5.827 Å, c=3.628 Å) and assuming one active site per Co-X_2 dumbbell (which translates into two active sites per square (111) face of one unit as shown below).

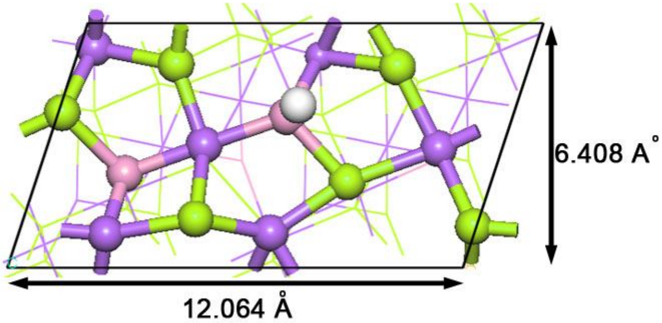

Therefore, the density of surface active sites of *o*-$CoSe_2$|P on geometric area is:

$$\frac{2}{12.064\times10^{-8}\ cm \times 6.408\times10^{-8}\ cm} \times 128 = 4.81 \times 10^{16}\ site\ \mathrm{cm^{-2}} \quad (2)$$

And the TOF per site at an overpotential of 104 mV (j = 10 mA cm^{-2}) is determined as follows:

$$\frac{0.01\ A}{1\ cm^2} \times \frac{1\ C}{1\ s*1\ \mathrm{A}} \times \frac{1\ mol}{96485\ C} \times \frac{6.02\times10^{23}\ e}{1\ mol} \times \frac{1}{2\ e} \times \frac{1\ cm^2}{4.81\times10^{16}\ atom} = 0.65\ \frac{H_2/s}{surface\ site} \quad (3)$$

Therefore, the TOF value of *o*-$CoSe_2$|P at η = 200 mV (j = 230 mA cm^{-2}) is determined as follows:

$$\frac{230\ mA\ cm^{-2}}{10\ mA\ cm^{-2}} \times 0.65 = 14.95 \frac{H_2/s}{surface\ site} \quad (4)$$

Similarly, the TOF of c-$CoSe_2$ could also be calculated by using the lattic parameter of cubic $CoSe_2$ (a=b=c=5.860 Å) and assuming active site is Co atom (which translates into three active sites per square 210 face of one unit as shown below):

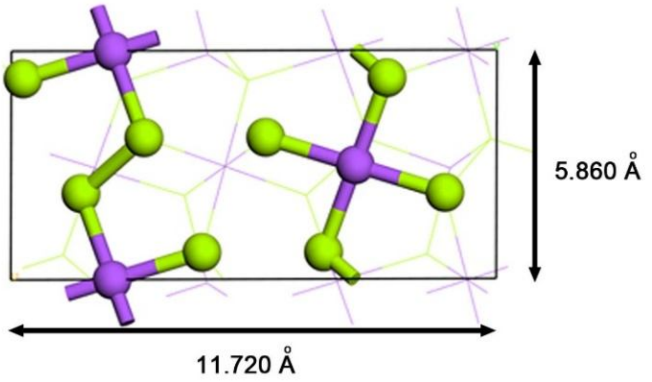

$$R_f(c - CoSe_2) = \frac{1.1\ \text{mF cm}^{-2}}{0.1\ \text{mF cm}^{-2}} = 10 \quad (5)$$

So the density of surface active sites of c-$CoSe_2$ on geometric area is:

$$\frac{3}{11.720\times10^{-8}\ cm\times5.86\times10^{-8}\ cm} \times 10 = 4.37 \times 10^{17}\ site\ \text{cm}^{-2} \quad (6)$$

Therefore, the TOF per site at an overpotential of 330 mV (j = 10 mA cm^{-2}) is determined as follows:

$$\frac{0.01\ A}{1\ cm^2} \times \frac{1\ C}{1\ s*1\ \text{A}} \times \frac{1\ mol}{96485\ C} \times \frac{6.02\times10^{23}\ e}{1\ mol} \times \frac{1}{2\ e} \times \frac{1\ cm^2}{4.37\times10^{17}\ atom} = 0.071\ \frac{H_2/s}{surface\ site} \quad (7)$$

Note that because the nature of the active sites and the real surface area of the o-$CoSe_2$|P and c-$CoSe_2$ electrocatalysts are difficult to determine accurately, the results provided here is just an indirect estimation.

Supplementary Note 2: Computational methods. The Gibbs free-energy (ΔG_{H*}) is expressed as: $\Delta G_{H*} = \Delta E_{H*} + \Delta E_{ZPE} - T\Delta S$. Where ΔE_{H*}, ΔE_{ZPE} and ΔS are the adsorption energy of atomic hydrogen on the given surface, zero point energy

correction and entropy change of H* adsorption, respectively. The zero point energy correction can be estimated by the equation $\Delta E_{ZPE} = E_{ZPE}(H^*)-1/2E_{ZPE}(H_2)$, where the $E_{ZPE}(H_2)$ is calculated to be 0.284 eV, in good agreement to the previous reports[58]. ΔS can be calculated by the equation $\Delta S = S(H^*)-1/2S(H_2) \approx -1/2S(H_2)$, due to the negligible of the entropy of hydrogen in adsorbed state. At 1 bar and 300 K, $T\Delta S$ is about -0.205 eV. ΔE_{H^*} is calculated as $\Delta E_{H^*} = E_{tot} - E_{sub} - 1/2E_{H2}$, where E_{tot} and E_{sub} are energies of H absorbed system and the clean given surface, and E_{H2} is the energy of H_2 molecular in gas phase.

Supplementary References

1 Liang, L. *et al.* Metallic Single-Unit-Cell Orthorhombic Cobalt Diselenide Atomic Layers: Robust Water-Electrolysis Catalysts. *Angew. Chem. Int. Ed.* **54**, 12004-12008 (2015).

2 Wu, R. J. *et al.* Atomic and electronic structure of exfoliated black phosphorus. *J. Vac. Sci. Technol. A* **33**, 060604 (2015).

3 Almeida, A. n. J. *et al.* Charge Trapping Defects in CdSe Nanocrystal Quantum Dots. *J. Phys. Chem. C* **120**, 13763-13770 (2016).

4 Azcondo, M. T. *et al.* Complex magnetic behaviour of $Sr_2CoNb_{1-x}Ti_xO_6$ ($0 \leqslant x \leqslant 0.5$) as a result of a flexible microstructure. *Dalton T.* **44**, 3801-3810 (2015).

5 Bollig, B., Geyzers, K. & Heuken, M. High‐resolution scanning transmission electron microscopy (STEM) and electron energy loss spectroscopy (EELS) of ZnS_xSe_{1-x}/ZnSe Quantum Wells. *Adv. Funct. Mater.* **3**, 183-190 (1994).

6 Saadi, F. H. *et al.* Operando Spectroscopic Analysis of CoP Films Electrocatalyzing the Hydrogen-Evolution Reaction. *J. Am. Chem. Soc.* **139**, 12927-12930 (2017).

7 Franke, R. & Hormes, J. The P *K*-near edge absorption spectra of phosphates. *Physica. B* **216**, 85-95 (1995).

8 Zhang, W. X. *et al.* A hydrothermal synthesis of orthorhombic nanocrystalline cobalt diselenide $CoSe_2$. *Mater. Res. Bull.* **35**, 2403-2408 (2000).

9 Gao, M. R., Yao, W. T., Yao, H. B. & Yu, S. H. Synthesis of Unique Ultrathin Lamellar Mesostructured $CoSe_2$-Amine (Protonated) Nanobelts in a Binary Solution. *J. Am. Chem. Soc.* **131**, 7486-7487 (2009).

10 Chen, P. Z. *et al.* Phase-Transformation Engineering in Cobalt Diselenide Realizing Enhanced Catalytic Activity for Hydrogen Evolution in an Alkaline Medium. *Adv. Mater.* **28**, 7527-7532 (2016).

11 Wu, Z. *et al.* Hierarchically Porous Electrocatalyst with Vertically Aligned Defect-Rich CoMoS Nanosheets for the Hydrogen Evolution Reaction in an Alkaline Medium. *ACS Appl. Mater. Inter.* **9**, 5288-5294 (2017).

12 Fang, L. *et al.* Tuning Unique Peapod-Like $Co(S_xSe_{1-x})_2$ Nanoparticles for Efficient Overall Water Splitting. *Adv. Funct. Mater.* **27**, 1701008 (2017).

13 Xu, K. *et al.* Regulating Water-Reduction Kinetics in Cobalt Phosphide for Enhancing HER Catalytic Activity in Alkaline Solution. *Adv. Mater.* **29**, 1606980 (2017).

14 Hu, C. & Dai, L. Multifunctional Carbon-Based Metal-Free Electrocatalysts for Simultaneous Oxygen Reduction, Oxygen Evolution, and Hydrogen Evolution. *Adv. Mater.* **29**, 1604942 (2017).

15 Xu, X. *et al.* A Perovskite Electrocatalyst for Efficient Hydrogen Evolution Reaction. *Adv. Mater.* **28**, 6442-6448 (2016).

16 Gao, X. *et al.* Hierarchical $NiCo_2O_4$ Hollow Microcuboids as Bifunctional Electrocatalysts for Overall Water-Splitting. *Angew. Chem. Int. Ed.* **55**, 6290-6294 (2016).

17 Jiang, J., Gao, M., Sheng, W. & Yan, Y. Hollow Chevrel-Phase $NiMo_3S_4$ for Hydrogen Evolution in Alkaline Electrolytes. *Angew. Chem. Int. Ed.* **55**, 15240-15245 (2016).

18 Shi, H., Liang, H., Ming, F. & Wang, Z. Efficient Overall Water-Splitting Electrocatalysis Using Lepidocrocite VOOH Hollow Nanospheres. *Angew. Chem. Int. Ed.* **56**, 573-577 (2017).

19 Vrubel, H. & Hu, X. Molybdenum boride and carbide catalyze hydrogen evolution in both acidic and basic solutions. *Angew. Chem. Int. Ed.* **51**, 12703-12706 (2012).

20 Stern, L.-A., Feng, L., Song, F. & Hu, X. Ni_2P as a Janus catalyst for water splitting: the oxygen evolution activity of Ni_2P nanoparticles. *Energy Environ. Sci.* **8**, 2347-2351 (2015)

21 Wu, H. B., Xia, B. Y., Yu, L., Yu, X.-Y. & Lou, X. W. Porous molybdenum carbide nano-octahedrons synthesized via confined carburization in metal-organic frameworks for efficient hydrogen production. *Nat. Commun.* **6**, 6512 (2015).

22 Feng, Y., Yu, X. Y. & Paik, U. Nickel cobalt phosphides quasi-hollow nanocubes as an efficient electrocatalyst for hydrogen evolution in alkaline solution. *Chem. Commun.* **52**, 1633-1636 (2016).

23 Tang, M. H. *et al.* Nickel-silver alloy electrocatalysts for hydrogen evolution and oxidation in an alkaline electrolyte. *Phys. Chem. Chem. Phys.* **16**, 19250-19257 (2014).

24 Xiao, P. *et al.* Molybdenum phosphide as an efficient electrocatalyst for the hydrogen evolution reaction. *Energy Environ. Sci.* **7**, 2624-2629 (2014).

25 Kwak, I. H. *et al.* $CoSe_2$ and $NiSe_2$ Nanocrystals as Superior Bifunctional Catalysts for Electrochemical and Photoelectrochemical Water Splitting. *ACS Appl. Mate Inter.* **8**, 5327-5334 (2016).

26 Ma, L., Ting, L. R. L., Molinari, V., Giordano, C. & Yeo, B. S. Efficient hydrogen evolution reaction catalyzed by molybdenum carbide and molybdenum nitride nanocatalysts synthesized via the urea glass route. *J. Mater. Chem. A* **3**, 8361-8368 (2015).

27 Song, B. *et al.* Tuning Mixed Nickel Iron Phosphosulfide Nanosheet Electrocatalysts for Enhanced Hydrogen and Oxygen Evolution. *ACS Catal.* **7**, 8549–8557 (2017).

28 Tian, J., Liu, Q., Asiri, A. M. & Sun, X. Self-supported nanoporous cobalt phosphide nanowire arrays: an efficient 3D hydrogen-evolving cathode over the wide range of pH 0-14. *J. Am. Chem. Soc.* **136**, 7587-7590 (2014).

29 Wang, X., Li, W., Xiong, D., Petrovykh, D. Y. & Liu, L. Bifunctional Nickel Phosphide Nanocatalysts Supported on Carbon Fiber Paper for Highly Efficient and Stable Overall Water Splitting. *Adv. Funct. Mater.* **26**, 4067-4077 (2016).

30 You, B., Jiang, N., Liu, X. & Sun, Y. Simultaneous H_2 Generation and Biomass Upgrading in Water by an Efficient Noble-Metal-Free Bifunctional Electrocatalyst. *Angew. Chem. Int. Ed.* **55**, 9913-9917 (2016).

31 Chen, P. *et al.* Phase-Transformation Engineering in Cobalt Diselenide Realizing Enhanced Catalytic Activity for Hydrogen Evolution in an Alkaline Medium. *Adv. Mater.* **28**, 7527-7532 (2016).

32 Liang, Y., Liu, Q., Asiri, A. M., Sun, X. & Luo, Y. Self-Supported FeP Nanorod Arrays: A Cost-Effective 3D Hydrogen Evolution Cathode with High Catalytic Activity. *ACS Catal.* **4**, 4065-4069 (2014).

33 Zhu, Y.-P., Liu, Y.-P., Ren, T.-Z. & Yuan, Z.-Y. Self-Supported Cobalt Phosphide Mesoporous Nanorod Arrays: A Flexible and Bifunctional Electrode for Highly Active Electrocatalytic Water Reduction and Oxidation. *Adv. Funct. Mater.* **25**, 7337-7347 (2015).

34 Zhang, R. *et al.* Ternary $NiCo_2P_x$ Nanowires as pH-Universal Electrocatalysts for Highly Efficient Hydrogen Evolution Reaction. *Adv. Mater.* **29,** 1605502 (2017).

35 Hou, Y. *et al.* Ternary Porous Cobalt Phosphoselenide Nanosheets: An Efficient Electrocatalyst for Electrocatalytic and Photoelectrochemical Water Splitting. *Adv. Mater.* **29,** 1701589 (2017).

36 Hou, Y. *et al.* Vertically oriented cobalt selenide/NiFe layered-double-hydroxide nanosheets supported on exfoliated graphene foil: an efficient 3D electrode for overall water splitting. *Energy Environ. Sci.* **9**, 478-483 (2016).

37 Liu, B. *et al.* Nickel-Cobalt Diselenide 3D Mesoporous Nanosheet Networks Supported on Ni Foam: An All-pH Highly Efficient Integrated Electrocatalyst for Hydrogen Evolution. *Adv. Mater.* **29**, 1606521 (2017).

38 Wang, W. Y. *et al.* A self-supported $NiMoS_4$ nanoarray as an efficient 3D cathode for the alkaline hydrogen evolution reaction. *J Mater. Chem. A* **5**, 16585-16589 (2017).

39 Feng, L. L. *et al.* High-index faceted Ni_3S_2 nanosheet arrays as highly active and ultrastable electrocatalysts for water splitting. *J. Am. Chem. Soc.* **137**, 14023-14026 (2015).

40 Sivanantham, A., Ganesan, P. & Shanmugam, S. Hierarchical $NiCo_2S_4$ Nanowire Arrays Supported on Ni Foam: An Efficient and Durable Bifunctional Electrocatalyst for Oxygen and Hydrogen Evolution Reactions. *Adv. Funct. Mater.* **26**, 4661-4672 (2016).

41 Zhang, J. *et al.* Interface Engineering of MoS_2/Ni_3S_2 Heterostructures for Highly Enhanced Electrochemical Overall-Water-Splitting Activity. *Angew. Chem. Int. Ed.* **55**, 6702-6707 (2016).

42 Liu, Y. *et al.* Coupling Sub-Nanometric Copper Clusters with Quasi-Amorphous Cobalt Sulfide Yields Efficient and Robust Electrocatalysts for Water Splitting Reaction. *Adv. Mater.* **29**, 1606200 (2017).

43 Laursen, A. B. *et al.* Nanocrystalline Ni_5P_4: a hydrogen evolution electrocatalyst of exceptional efficiency in both alkaline and acidic media. *Energy Environ. Sci.* **8**, 1027-1034 (2015).

44 Jia, Y. *et al.* A Heterostructure Coupling of Exfoliated Ni-Fe Hydroxide Nanosheet and Defective Graphene as a Bifunctional Electrocatalyst for Overall Water Splitting. *Adv. Mater.* **29**, 1700017 (2017).

45 Luo, J. *et al.* Water photolysis at 12.3% efficiency via perovskite photovoltaics and Earth-abundant catalysts. *Science* **345**, 1593-1596 (2014).

46 Jin, H. *et al.* In situ cobalt-cobalt oxide/N-doped carbon hybrids as superior bifunctional electrocatalysts for hydrogen and oxygen evolution. *J. Am. Chem. Soc.* **137**, 2688-2694 (2015).

47 Zhu, Y. P., Ma, T. Y., Jaroniec, M. & Qiao, S. Z. Self-Templating Synthesis of Hollow Co_3O_4 Microtube Arrays for Highly Efficient Water Electrolysis. *Angew. Chem. Int. Ed.* **56**, 1324-1328 (2017).

48 Gong, M. *et al.* Nanoscale nickel oxide/nickel heterostructures for active hydrogen evolution electrocatalysis. *Nat. Commun.* **5**, 4695 (2014).

49 Wang, H. *et al.* Bifunctional non-noble metal oxide nanoparticle electrocatalysts through lithium-induced conversion for overall water splitting. *Nat. Commun.* **6**, 7261 (2015).

50 Zhang, H. *et al.* Active Sites Implanted Carbon Cages in Core-Shell Architecture: Highly Active and Durable Electrocatalyst for Hydrogen Evolution Reaction. *ACS Nano* **10**, 684-694 (2016).

51 Zou, X. *et al.* Cobalt-embedded nitrogen-rich carbon nanotubes efficiently catalyze hydrogen evolution reaction at all pH values. *Angew. Chem. Int. Ed.* **53**, 4372-4376 (2014).

52 Hou, Y. *et al.* Efficient Electrochemical and Photoelectrochemical Water Splitting by a 3D Nanostructured Carbon Supported on Flexible Exfoliated Graphene Foil. *Adv. Mater.* **29**, 1604480 (2017).

53 Zhang, J. & Dai, L. Nitrogen, Phosphorus, and Fluorine Tri-doped Graphene as a Multifunctional Catalyst for Self-Powered Electrochemical Water Splitting. *Angew. Chem. Int. Ed.* **55**, 13296-13300 (2016).

54 Yu, Z. Y. *et al.* A one-dimensional porous carbon-supported Ni/Mo_2C dual catalyst for efficient water splitting. *Chem. Sci.* **8**, 968-973 (2017).

55 McKone, J. R., Sadtler, B. F., Werlang, C. A., Lewis, N. S. & Gray, H. B. Ni–Mo nanopowders for efficient electrochemical hydrogen evolution. *ACS Catal.* **3**, 166-169 (2013).

56 Zhang, J. *et al.* Efficient hydrogen production on $MoNi_4$ electrocatalysts with fast water dissociation kinetics. *Nature Commun.* **8**, 15437 (2017).

57 Zhuo, J. Q. *et al.* High-Performance Electrocatalysis for Hydrogen Evolution Reaction Using Se-Doped Pyrite-Phase Nickel Diphosphide Nanostructures. *ACS Catal.* **5**, 6355-6361 (2015).

58 Norskov, J. K. *et al.* Trends in the exchange current for hydrogen evolution. *J. Electrochem. Soc.* **152**, 23-26 (2005).
